# From multi-omics approaches to personalized medicine in myocardial infarction

**DOI:** 10.3389/fcvm.2023.1250340

**Published:** 2023-10-30

**Authors:** Chaoying Zhan, Tong Tang, Erman Wu, Yuxin Zhang, Mengqiao He, Rongrong Wu, Cheng Bi, Jiao Wang, Yingbo Zhang, Bairong Shen

**Affiliations:** ^1^ Department of Cardiology and Institutes for Systems Genetics, Frontiers Science Center for Disease-Related Molecular Network, West China Hospital, Sichuan University, Chengdu, China; ^2^ Key Laboratory of Bio-Resource and Eco-Environment of Ministry of Education, College of Life Sciences, Sichuan University, Chengdu, China; ^3^ Tropical Crops Genetic Resources Institute, Chinese Academy of Tropical Agricultural Sciences, Haikou, China

**Keywords:** myocardial infarction, multi-omics, personalized medicine, single-omics, integrative analysis

## Abstract

Myocardial infarction (MI) is a prevalent cardiovascular disease characterized by myocardial necrosis resulting from coronary artery ischemia and hypoxia, which can lead to severe complications such as arrhythmia, cardiac rupture, heart failure, and sudden death. Despite being a research hotspot, the etiological mechanism of MI remains unclear. The emergence and widespread use of omics technologies, including genomics, transcriptomics, proteomics, metabolomics, and other omics, have provided new opportunities for exploring the molecular mechanism of MI and identifying a large number of disease biomarkers. However, a single-omics approach has limitations in understanding the complex biological pathways of diseases. The multi-omics approach can reveal the interaction network among molecules at various levels and overcome the limitations of the single-omics approaches. This review focuses on the omics studies of MI, including genomics, epigenomics, transcriptomics, proteomics, metabolomics, and other omics. The exploration extended into the domain of multi-omics integrative analysis, accompanied by a compilation of diverse online resources, databases, and tools conducive to these investigations. Additionally, we discussed the role and prospects of multi-omics approaches in personalized medicine, highlighting the potential for improving diagnosis, treatment, and prognosis of MI.

## 1. Introduction

Cardiovascular disease (CVD) is a leading cause of mortality globally, responsible for nearly half of all deaths. Among various types of CVD, myocardial infarction (MI), is the main cause of cardiovascular death and one of the most common types of coronary artery disease (CAD) (1). In recent years, MI have been occurring in increasingly younger individuals due to changes in lifestyle, unclear circadian rhythms, and increased social pressure (2, 3). MI is characterized by irreversible myocardial necrosis caused by coronary artery ischemia and hypoxia. After MI occurs, a large number of fibroblasts replace necrotic cardiomyocytes, leading to ventricular remodeling such as myocardial fibrosis and cardiac hypertrophy (4). These changes can ultimately lead to adverse events such as cardiac rupture, heart failure (HF), and sudden death due to insufficient cardiac motility (5–7). Although methods such as bypass grafting, percutaneous coronary intervention (PCI), and antithrombotic drugs are available for MI treatment, they can only reduce the severity of CAD to a certain extent. Due to the complexity and diversity of the disease, these treatments cannot reverse myocardial necrosis and ventricular remodeling caused by ischemia and hypoxia (4). Therefore, it remains the focus of research to elucidate the diverse molecular mechanism of MI and to find efficient markers, which is of great significance to improve the diagnosis, treatment effect and prognosis of MI.

Since the turn of the century, the completion and deepening of the Human Genome Project (HGP) (8) and the Encyclopedia of DNA Elements (ENCODE) (9) have established a robust foundation for personalized medicine research and the investigation of the pathogenesis of complex diseases. In recent years, the wide application of high-throughput technology and high-resolution mass spectrometry (HRMS) has not only increased the amount of biological data on the molecular mechanisms of diseases but also expanded the dimension of such data. This has encouraged researchers to explore the molecular mechanisms of diseases using multi-omics technologies and methods. Diseases, including MI, are now understood more comprehensively at various molecular levels, including genomics, epigenetics, transcriptomics, proteomics, metabolomics, etc.

**Figure 1:**
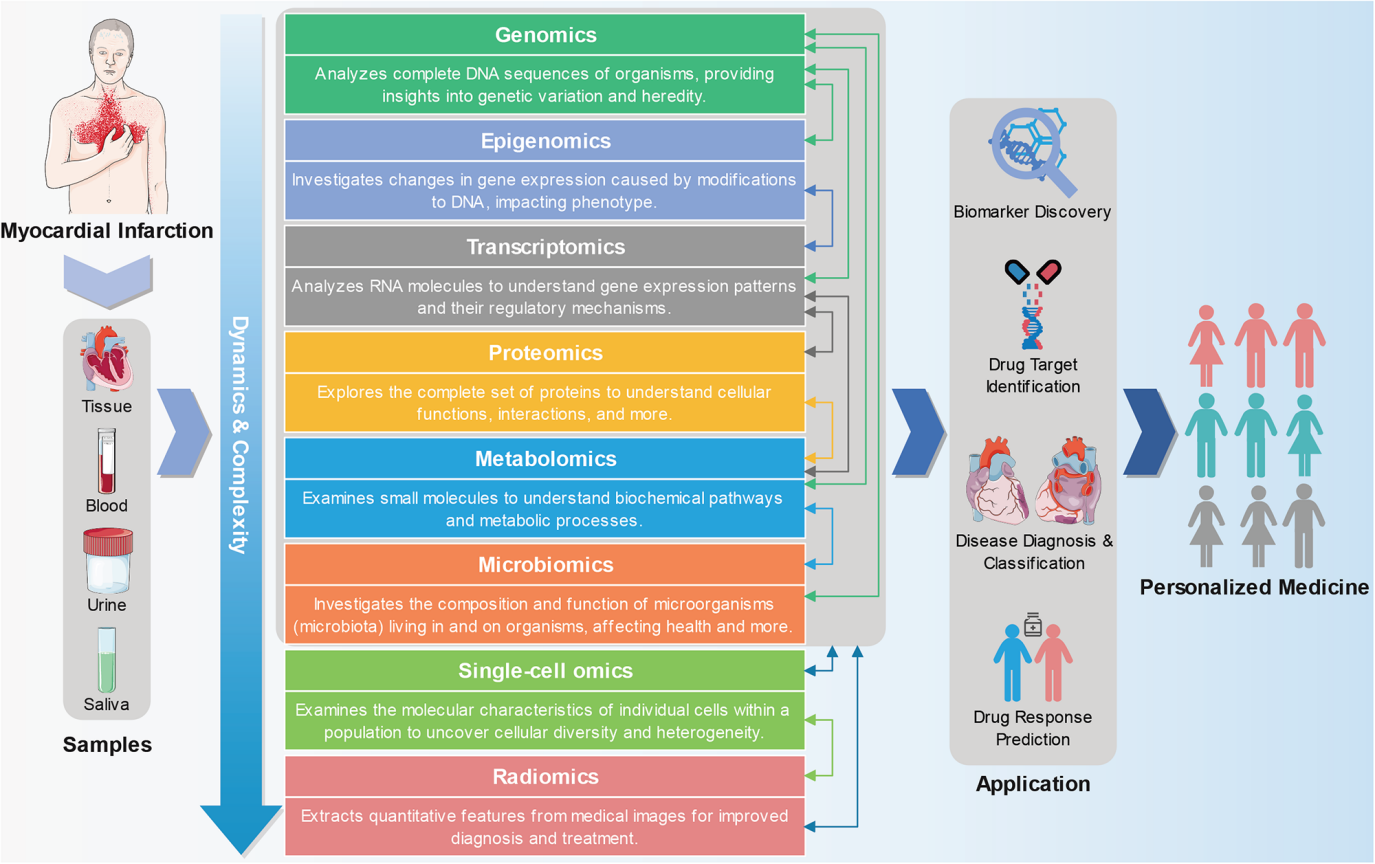
From multi-omics approaches to personalized medicine in myocardial infarction. Genomics, epigenomics, transcriptomics, proteomics, metabolomics, and other omics technologies can be used to detect and analyze tissue or body fluid (such as blood, urine, and saliva) samples from MI patients. Omics and multi-omics approaches offer a wide range of applications in advancing precision medicine, encompassing biomarker discovery, drug target identification, patient stratification, and predicting drug responses. The part of the figure is drawn with the pictures of Servier Medical Art (https://smart.servier.com).

MI is a complex disease caused by environmental and genetic factors, and its various subtypes exhibit different pathogenesis and prognoses (10). While some progress has been made in single-omics studies of MI over the past few decades, the pathogenesis of MI remains unclear. The development of multi-omics methods is expected to shed light on the molecular pathogenesis and differences among various MI subtypes, identify biomarkers with diagnostic, therapeutic, and prognostic values, and ultimately enable the prediction, prevention, and personalized treatment of MI. Therefore, this study summarized the research progress made in MI research across various omics fields, including genomics, epigenomics, transcriptomics, proteomics, metabolomics, and others. In particular, we emphasized the significance and potential of multi-omics approaches in realizing personalized medicine for MI (Figure 1).

## 2. Single-omics approaches

### 2.1. Genomics

Genomics is a field of study that encompasses the systematic analysis of all genes in an organism. The development of genome sequencing technologies, including Sanger sequencing (11), DNA microarray (12), and next-generation sequencing (NGS) (13). Of these technologies, DNA microarrays and NGS are widely used for studying gene mutations, which can help identify candidate genes associated with diseases and analyze genotype sensitivity to drugs. Gene mutations can be broadly categorized into three types: single nucleotide variations (SNVs), insertions/deletions (In/Dels), and copy number variations (CNVs). These types of mutations can contribute to the development of various diseases, including MI. Therefore, genomics plays an important role in elucidating the genetic basis of diseases and developing personalized medicine.

Researchers have identified numerous gene associated with an increased risk of MI, offering crucial insights into a deeper understanding of the genetic basis of this heart disease. Among these, genes in the 9p21.3 region have been identified as important genetic risk factors for MI. Variants in this genetic region, including *CDKN2B/CDKN2B-AS1* rs1333049 (14, 15), *MTAP* rs7027989 (16), and *ANRIL* rs9632884 (17), were significantly associated with an elevated risk of MI. The C risk allele and CC genotype of rs1333049 were both linked to a higher risk of not only MI but also other CVDs (14, 15, 18, 19). The renin-angiotensin system is crucial regulating blood pressure and the development of CAD. Studies have found that polymorphisms in renin-angiotensin system-related genes, including *AGT* rs4762 (20), *AGTR1* rs5186 (21), *AGTR2* rs11091046 (22), *KLK1* rs5517 (23), *ACE* rs1799752 (24), as well as *ACE2 rs4646142 and rs1978124* (25), were associated with the risk of MI*.* The *ACE* gene insert/delete (I/D, rs1799752) polymorphism is the most extensively studied. This polymorphism refers to the existence or deletion of an Alu repeat sequence of 287 base pairs in intron 16 of the *ACE* gene, which can alter the activity of the angiotensin-converting enzyme (ACE) protein and lead to enhanced plaque vulnerability, ulceration, and thrombosis, ultimately leading to MI (26). Numerous studies have shown that the DD genotype of the *ACE* gene is not only a risk factor for MI (23, 27–29) but also related to the poor prognosis (30–32). The coagulation and fibrinolytic systems play pivotal roles in thrombus formation and dissolution. Abnormal mutations in specific coagulation factors, such as fibrinogen (*FGA*, *FGB*, *FGG)* (33), *F2* (34), *F5* (34), *F7* (35), *VWF* (36), as well as genes in the fibrinolytic system like *PLAT* (37), and *SERPINE1* (38), can lead to abnormal thrombosis or the formation of thrombi that are challenging to dissolve, subsequently increasing the risk of MI. Furthermore, genetic variants related to lipid metabolism and the inflammatory response are closely associated with the risk of MI, such as *APOE* rs7412 (39), *CETP* rs429358 (39), *LPL* rs328 (40), *IL-6* rs1800795 (41), and *TNF* rs1800629 (42). These genetic variations can influence multiple biological processes, including cholesterol metabolism, vascular inflammation, plaque formation, and more, thereby increasing the risk of MI (43–45). The identification of these genetic risk factors not only helps uncover the genetic basis of MI but also offers new opportunities for disease prediction and prevention. Based on known genetic risk factors, researchers have developed genetic risk scoring systems that can estimate an individual's risk of MI (46–48). This personalized risk assessment aids medical professionals in better identifying high-risk patients and initiating appropriate interventions, including lifestyle modifications and medications, to reduce the risk of MI.

Genomics technology can be employed to identify genetic markers that can predict an individual's response to drugs. Clopidogrel is an antiplatelet drug commonly used for anticoagulant therapy in patients with MI. However, some individuals respond poorly to clopidogrel, which may be related to *CYP2C19* loss-of-function mutations that slow drug metabolism and thus reduce the drug's efficacy (49, 50). Warfarin is a commonly used anticoagulant drug for treating patients with heart disease. Individual warfarin dosage requirements vary based on polymorphisms *1, *2 and *3 for *CYP2C9*, −1639G > A for *VKORC1* allowing for a more accurate determination of the drug dosage to prevent issues such as bleeding or insufficient clotting (51, 52). Angiotensin modulators refer to a class of drugs that interact with the renin-angiotensin-aldosterone system in the body to regulate blood pressure and fluid balance, including ACE inhibitors and angiotensin II receptor blockers (ARBs). The *ACE* I/D polymorphism, *AGT* rs7079, and *AGTR1* haplotypes have been proven to be associated with variable responses to angiotensin modulators, impacting both neurological outcomes and blood pressure variations (53, 54). Orbofiban is a medication that belongs to a class of drugs known as platelet aggregation inhibitors or antiplatelet agents. MI patients with the T allele of the *GNB3* gene were more likely to experience bleeding when given orbofiban, indicating that genetics can influence the risk of bleeding with antiplatelet drugs (55). These genetic markers can help doctors better select appropriate drugs and dosages to increase the effectiveness of treatment and reduce the risk of adverse reactions.

### 2.2. Epigenomics

Epigenomics is a specialized branch of genomics that focuses on the comprehensive analysis of epigenetic changes at the genomic level, including DNA methylation, histone modification, and chromatin structure, across the entire genome of an organism or a specific cell type (56). These epigenetic changes play a critical role in the development and progression of human diseases, including MI. By identifying the specific epigenetic changes associated with MI, researchers can identify potential therapeutic targets and develop personalized treatment strategies for patients.

DNA methylation is an epigenetic marker that can be inherited, and it involves the transfer of a methyl group to the cytosine 5 carbon site through DNA methyltransferase. This process typically occurs in the promoter region and serves to inhibit gene transcription. Abnormal hypermethylation, which can lead to transcriptional silencing, is often associated with diseases and can be used as a biomarker (57). Recent studies have found that DNA methylation plays an important role in the development of MI. DNA methylation patterns differ between patients with MI and healthy individuals (58). These aberrant methylation patterns can result in altered gene expression, influencing the onset and progression of MI. GPR15 gene may contribute to MI by influencing inflammation and angiogenesis-related pathways (59, 60), with DNA hypomethylation in this gene associated with an increased risk of early-onset MI due to the upregulation of *GPR15* expression levels (61). ABO blood types are linked to the risk of MI, with non-blood type O individuals having a higher risk (62, 63). The DNA methylation of the *ABO* gene promoter plays a pivotal role in regulating *ABO* gene expression (64), where increased methylation status of the *ABO* gene promoter was associated with an elevated risk of AMI by suppressing *ABO* mRNA expression (65, 66). An epigenome-wide association study identified 34 differentially methylated CpG sites associated with MI. These CpGs may contribute to MI through influencing the expression of genes related to inflammatory and lipid metabolism, including *MPO, SERPINA1, NISCH, DLEU1, ZFPM1*, and others (67). Furthermore, biomarkers derived from DNA methylation data, such as GrimAgeAccel, PhenoAgeAccel, EEAA, and DNAmRS, can predict the risk of MI. Among these, GrimAgeAccel has proven to be the most effective tool for evaluating MI risk (68).

DNA methylation is an emerging and exciting research field in the treatment of MI, which is still in the early stages of exploration and development. DNA methylation can affect the expression and activity of mitochondria, antioxidant and apoptosis genes, thereby leading to ischemia-reperfusion (I/R) injury (69). I/R injury plays a key role in the process of MI (70), so a deep understanding of the role of DNA methylation in this process is crucial for developing targeted therapeutic strategies. Some studies have begun to explore drug intervention in DNA methylation to improve the recovery of patients with MI. These drugs can affect the methylation status of specific genes and are expected to promote cardiac repair and regeneration after MI. DNA methyltransferase-1 (DNMT1) has been identified as a key player in cardiac fibrosis by regulating miR-133b methylation, affecting myofibroblast activation and *CTGF* expression. 5-Azacytidine can counteract DNMT1-induced miR-133b methylation, delaying myocardial fibrosis (71). Additionally, *APAF1* gene is a key player in the regulation of apoptosis, a fundamental process in cell biology (72). DNMT1 has also been identified as playing a role in the methylation of the *APAF1* promoter, silencing the *APAF1* gene. This mechanism reinforces the protective effect of sevoflurane against cardiomyocyte injury induced by hypoxia/reoxygenation (73). Long non-coding RNA (lncRNA) ZFAS1 has been demonstrated to be upregulated during cardiac I/R injury, affecting cardiac function by influencing the methylation level of the *Notch1* gene. Nicotinamide mononucleotide could enhance *Notch1* expression, leading to improved cardiomyocyte survival and cardiac function (74). Furthermore, lifestyle factors, such as diet, exercise, and smoking cessation, have been demonstrated to influence DNA methylation (75–77). Maintaining a healthy lifestyle during MI recovery can potentially enhance cardiovascular health by impacting DNA methylation. Healthcare professionals play a crucial role in mitigating the risk of MI by educating patients about making healthy lifestyle choices.

Histones are essential for maintaining the shape and structure of chromatin, and the tail region of histones undergoes various post-translational modifications such as methylation, acetylation, and phosphorylation. These modifications can lead to changes in chromatin conformation, ultimately affecting the transcription of important genes (78, 79). Histone methylation, particularly trimethylation of histone H3 on lysine-4 (H3K4me3), plays a crucial role in MI (80). JMJD3 demethylase was found to exacerbate cardiac fibrosis by reducing H3K27me3 at the beta-catenin promoter in activated cardiac fibroblasts, worsening cardiac fibrosis. Inhibiting JMJD3 may be a potential therapy for cardiac fibrosis (81). Another study showed that *Salvia miltiorrhiza* and *Carthamus tinctorius* extract (SCE) effectively reduced myocardial fibrosis and inflammation by inhibiting H3K4me3 and H3K36 trimethylation (H3K36me3) at the *Smad3* promoter in cardiac fibroblasts, leading to decreased Smad3 transcription (82). Additionally, riboflavin has been shown to protect myocardium from damage by regulating phospholipid metabolism and H3K4me2 (83). Histone acetyltransferases (HATs) and histone deacetylases (HDACs) play crucial roles in regulating histone acetylation, which in turn affects inflammation, myocardial function, and cardiac repair following MI (84, 85). After a MI, miRNA-134-5p goes up, inhibiting histone H3K14 acetylation by lowering lysine acetyltransferase 7 expression. This reduces antioxidant enzyme activity, raising oxidative stress and promoting harmful heart remodeling (86, 87). Eicosapentaenoic acid, docosahexaenoic acid, and ecklonia stolonifera Okamura extract were found to inhibit p300 HAT activity. This inhibition reduced histone acetylation and regulates gene expression, ultimately suppressing cardiomyocyte hypertrophy and preventing HF development (88, 89). Sodium caprylate has been shown to enhance cardiac recovery after MI by acting on HAT Kat2a, increasing histone acetylation levels, activating antioxidant gene expression, and reducing cardiomyocyte apoptosis (90). Studies have shown that inhibition of HDAC1 (91), HDAC3 (92), HDAC4 (93), HDAC5 (94), HDAC6 (95), and HDAC9 (96) has a protective effect on cardiomyocyte apoptosis, left ventricular remodeling (LVR), cardiac disfunction, and cardiac fibrosis after MI. This signifies the potential therapeutic value of targeting these specific HDACs to enhance post-MI cardiac recovery and mitigate adverse remodeling processes. Therefore, studying histone modifications is expected to offer valuable insights for developing new treatment strategies to alleviate MI and promote patient recovery.

### 2.3. Transcriptomics

Transcriptomics refers to the study of all RNA transcripts within a specific species, including both mRNA and non-coding RNA (ncRNA). Gene transcription is known to be spatiotemporal specific, meaning that gene expression can vary between different tissues or at different stages of the same tissue. The analysis of transcription plays a critical role in identifying the structure and function of the genome, decoding the genetic network underlying diseases, and searching for sensitive molecular biomarkers for diseases, drugs, and pathogens (97, 98).

Transcriptomic studies have identified many genes associated with MI, including genes that are up- or down-regulated after MI. These differentially expressed genes can reveal changes in cell signaling pathways after infarction and possible therapeutic targets. Sheng et al. (99) identified 552 differentially expressed genes and 23 differentially expressed lncRNAs of MI by analyzing the gene expression profile of circulating endothelial cells. They observed that inflammation-related genes such as *NR4A2*, *IRAK3*, *NFIL3*, *IL1R2*, *CLEC4E* and *BCL3AMI* were highly up-regulated, indicating that inflammation is an important feature of MI. Another study employed a bioinformatic analysis to pinpoint eight key immuno-inflammation-related genes, namely *SH2D1B*, *ADM*, *PI3*, *MMP9*, *NRG1*, *CBLB*, *RORA*, and *FASLG*, which have been identified as potential biomarkers for AMI (100). Zhuo et al. (101) constructed an MI-related lncRNA-miRNA-mRNA network using RNA sequencing data and identified lncRNA SNHG8, hsa-miR-411-5p, *SOCS3* and *ICAM1* were key nodes in the network. Their findings demonstrated that lncRNA SNHG8 not only emerged as a risk factor for MI but also exhibited substantial diagnostic potential. Furthermore, extensive transcriptomic studies have revealed a significant correlation between the abnormal expression of inflammation-related genes, such as *VEGFA* (102), *TNF* (103), *IL6* (103, 104), *IL6R* (104), *PTGS2* (105), immune response-related genes including *CDKN2B* (106), *CDKN1C* (107), *TLR2* (108), *TLR4* (108), apoptosis and proliferation-related genes like *F3* (109), *BTG2* (110), *TXNIP* (111), *SAMSN1* (112), lipid metabolism-related genes such as *PPARGC1A* (113), *ACSL1* (114), *ABCG1* (115), *SULT2B1* (116), and extracellular matrix (ECM) and collagen-related genes, such as *MMP2* (117), *MMP9* (117), *LTBP4* (118), *TNXB* (118), and the occurrence and development of MI. These findings offer crucial insights into a deeper comprehension of the mechanisms underlying MI and hold promise for the development of more precise approaches for preventing and treating heart disease in the future.

NcRNAs, including microRNAs (miRNAs), lncRNAs and circular RNAs (circRNAs), have a significant attention in the field of MI research. They not only contribute to a deeper understanding of the disease's pathogenesis but also serve as valuable biomarkers, diagnostic tools, prognostic indicators, and potential therapeutic targets, offering new hope for managing MI patients (119–123). Numerous studies have demonstrated significant changes in the expression profiles of ncRNAs in both cardiac tissue and body fluids after MI. Some ncRNAs are upregulated, while others are downregulated. These changes are involved in the regulation of crucial pathological processes such as inflammation, apoptosis, and fibrosis, aiding in a more profound comprehension of the molecular basis of this disease (121, 123–125). Furthermore, many ncRNAs have been identified as biomarkers that can be utilized for MI diagnosis. These include miRNAs such as miRNA-1-3p (126, 127), miRNA-208a-3p (127, 128), miRNA-499a-5p (127), miRNA-486-5p (129), miRNA-21-5p (129, 130), as well as lncRNAs such as N1LR (131), SNHG1 (131), HIF1A-AS2 (132), TTTY15 (133), HULC (133), and circRNAs like cZNF292 (134), circTMEM165 (135), circUBAC2 (135), circZNF609 (135), circANKRD12 (135), circSLC8A1 (135), and among others. Additionally, ncRNAs are also employed to assess patients' prognosis, assist in determining treatment strategies, and predict disease progression. They are associated with patients' cardiac function, the risk of recurrent MI, and the occurrence of adverse events (124, 136–140). This information can offer valuable assistance to healthcare professionals in making well-informed decisions regarding treatment and predicting patient outcomes.

Transcriptome research plays a crucial role in treating MI. By studying how genes are active in heart tissue or cells, transcriptome research uncovers how drugs affect the body's molecular responses. This helps us understand treatment effects and mechanisms better, leading to more personalized therapies. In rat studies, it was indicated that photobiomodulation therapy can intervene in the activation of cardiac fibrosis after MI by altering gene activities and miRNAs in heart (141, 142). Su et al. (143) analyzed gene and ncRNA expression to uncover potential molecular mechanisms linked to the varying effectiveness of acubitril/valsartan treatment in patients with HF after AMI. These type of studies offers valuable insights into a deeper comprehension of drug mechanisms of action and resistance. Transcriptome research also aids in identifying potential biomarkers that can be utilized to assess treatment outcomes, monitor treatment progression, and predict patient responses to therapy. For instance, plasma levels of miR-223 and miR-126 have demonstrated potential as predictive biomarkers of dual antiplatelet therapy response and prognosis in patients with ST-segment elevation MI (STEMI) (144). Furthermore, abundant research suggests that miRNA hold promising potential in treating MI by regulating gene expression and crucial cellular processes. Key therapeutic candidates include miRNA-21 (145–147), miRNA-192-5p (148), and miRNA-432-5p (148), involved in inflammation and fibrosis; miRNA-499 (149), implicated in endothelial injury; miRNA-126 (150, 151), contributing to angiogenesis; miRNA-133 (152), influencing cardiac function; and miRNA-208a (153), linked to fibrosis. Intervening with these miRNAs could fundamentally change MI treatment by adjusting core processes and facilitating cardiac recovery.

### 2.4. Proteomics

Proteomics studies all proteins in tissues or cells, including protein expression levels, post-translational modifications, and protein-protein interactions to the study structure and location of protein and protein-protein interactions, providing a direct basis for clarifying the nature of life phenomena. Commonly used proteomics technologies include mass spectrometry (MS), two-dimensional gel electrophoresis, protein microarrays, protein-protein interaction assays, and imaging techniques like fluorescence and electron microscopy (154). Proteomics has played a crucial role in improving our understanding of the molecular mechanisms underlying MI and identifying potential protein markers of this disease.

Proteomics has unveiled dynamic fluctuations in protein expression during MI. By analyzing proteins in myocardial samples obtained from MI patients and animal models, scientists have identified a variety of proteins associated with MI, including inflammatory mediators, apoptosis-related proteins, and cardiac contractile proteins, shedding light on multiple key pathways involved in the development of MI (155–159). For example, Das et al. (160) used Orbitrap MS to identify 38 up-regulated and 26 down-regulated proteins in MI patients, most of which were related to chronic inflammation, atherosclerosis, and cholesterol reverse transport. Pan et al. (161) used ITRAQ (Isobaric tags for relative and absolute quantification) combined with LC-MS/MS (Liquid chromatography-tandem mass spectrometer) technologies to identify 95 MI differential expression proteins related to carbon metabolism, toll-like receptor signal pathway and hypertrophic cardiomyopathy. The proteomics approach has led to the discovery of an increasing number of diagnostic biomarkers, such as plasminogen (162), complement C8 beta chain (162), coagulation factor II (162), alpha-1 acid glycoprotein 2 (163), corticosteroid-binding globulin (163), serotransferrin (163), lactate dehydrogenase (164), creatine kinase (164), haptoglobin (165), etc. In a mouse model, researchers conducted proteomic analysis and identified cardiac myosin binding protein-C (cMyBP-C) as a potential up-regulated biomarker for MI (166). The potential of cMyBP-C as a sensitive cardiac-specific biomarker of MI was further confirmed by measuring its increased levels in the plasma of MI patients through enzyme-linked immunosorbent assay (ELISA) (167). Furthermore, proteomic approaches have identified many potential biomarkers for predicting MI prognosis by revealing distinct protein expression patterns. By examining the distinct protein expression patterns associated with MI, researchers have been able to discover valuable biomarkers that can provide insights into various outcomes, including HF (168), pulmonary hypertension (169), LVR (170), chronic kidney disease (171), and long-term outcomes (171). Liu et al. (168) found NF-*κ*B signaling-related proteins linked to HF after MI. Another study also identified 50 proteins during MI patient hospitalization for predicting long-term HF occurrence (172). These proteins hold promise as potential markers for diagnosing HF after MI. LVR is a prevalent complication following MI, and researchers have identified several potential protein biomarkers associated with this condition. These biomarkers include apolipoprotein A1, immunoglobulin A, interleukin-17E, tissue inhibitor of metalloproteinases-1, urokinase-type plasminogen activator, midkine, proprotein convertase subtilisin/kexin type 6, among others (170, 173, 174). Furthermore, proteomic studies can provide useful information in assessing left ventricular ejection fraction (LVEF) and infarct size after MI (175).

Currently, established cardiac biomarkers such as cardiac troponin (cTn), creatine kinase (CK), creatine kinase MB (CK-MB), copeptin, and heart-type fatty acid binding protein (H-FABP) have been validated as valuable for the diagnosis and prognosis of MI (176, 177). Among these, cTn is acknowledged as a biomarker for assessing the risk of acute coronary syndrome (ACS), including cardiac troponin T (cTnT) and cardiac troponin I (cTnI) (178). Furthermore, protein biomarkers can provide significant diagnostic information for MI, which is crucial for follow-up treatment. For instance, myeloid-related protein 8/14 (MRP-8/14) was found to have higher concentrations, and high-sensitivity cardiac troponin I (hs-cTnI) to have lower concentrations in type 2 MI compared to type 1 MI (179). Pandey et al. (180) demonstrated that the levels of cTnT and CK-MB were significantly higher in patients with type 1 MI than in those with type 2 MI, and cTnT increased disproportionately with CK-MB in type 2 MI patients. Additionally, coagulation factor VII levels were significantly higher in patients with silent MI than in those with clinical MI in another study (181).

Proteomics analysis can be employed to unravel the complex mechanisms underlying the therapeutic effects of various interventions in the context of MI. Within the domain of MI and its treatment, proteomic studies have illuminated the cardioprotective effects of different compounds. For example, a proteomic study demonstrated that the cardioprotective effects of Shexiang Baoxin pill (SBP) and Suxiao Jiuxin pill (SJP) in MI rats are achieved by modulating pathways associated with focal adhesion, and platform activation (182). Additionally, SBP's cardioprotective mechanisms were also found to involve energy metabolism within cardiac tissue (183). Wang et al. (184) employed proteomics to delve into the therapeutic effects of Salviae Miltiorrhizae and Cortex Moutan extract post-MI in rats. Their findings underscored the significance of metabolism, oxidative stress, and cytoskeleton modulation in these effects. Proteomics has provided insights into the regulatory protein targets impacted by specific treatments, offering valuable knowledge for advancing therapeutic strategies. Through the utilization of the SOMAScan aptamer-proteomics platform, George et al. (185) identified a cluster of five proteins whose regulation is influenced by the antagonistic effects of IL-6. These identified proteins were speculated to potentially play a role in mediating the therapeutic effects of tocilizumab in cases of non-ST segment elevation MI (NSTEMI). Furthermore, proteomic investigations have unveiled the positive influence of physical exercise on cardiac remodeling after MI. These benefits are attributed to increased anti-oxidant levels, diminished ion channel expression, favorable adaptations in energy metabolism, dampened inflammation, and alterations in ECM organization (186, 187).

### 2.5. Metabolomics

Metabolomics is a research method used to quantitatively analyze all metabolites in an organism and investigate the relationship between metabolites and physiological and pathological changes. This method employs various technologies, such as nuclear magnetic resonance (NMR) spectroscopy, MS, gas chromatography (GC), liquid chromatography (LC), capillary electrophoresis (CE), Fourier transform infrared (FTIR) spectroscopy, and Raman spectroscopy to study the small molecules produced by metabolic processes in biological fluids, cells, and tissues (188). Metabolomics studies have identified several dysregulated metabolites during MI, including lipids, amino acids, energy metabolites, and oxidative stress markers (189–191).

Research has revealed notable distinctions in metabolite profiles between patients with MI and healthy individuals or other populations (192, 193). Some metabolites, such as 12,13-diHOME, noradrenaline, tryptophan, and cysteic acid, were significantly elevated in patients with MI, while some antioxidants like glutathione were decreased (194–196). Changes in these metabolites may serve as potential biomarkers of MI. Abnormal amino acid metabolism is one of the early indicators of MI. Research has shown that amino acids and their metabolites such as tryptophan, carnitine, l-homocysteine sulfinic acid, kynurenine, and cysteic acid, significantly increase in the serum after MI, while amino acids like leucine, isoleucine, l-proline, l-alanine, glycine, l-cysteine, and l-cysteine sulfinic acid decrease (190, 195, 197). These changes in amino acid concentrations can serve as biomarkers for MI, aiding in early diagnosis and monitoring of patients' conditions. Lipid metabolism also undergoes significant changes in MI. Triglycerides, low-density lipoprotein cholesterol (LDL-C), non-high-density lipoprotein cholesterol (non-HDL-C), remnant cholesterol, and total cholesterol levels significantly increase after MI, while high-density lipoprotein cholesterol (HDL-C) decreases (198–201). This lipid metabolism abnormality is closely associated with the development of coronary artery atherosclerosis and can be used to predict the risk of MI. MI results in an insufficient energy supply to myocardial cells. Therefore, metabolomics research places a significant emphasis on metabolites related to energy metabolism. Examples of such metabolites include lactic acid (202), fatty acids (203), phosphates (204, 205), and creatine (206), all of which experience alterations following MI and can serve as biomarkers for this condition. Additionally, MI is characterized by disruptions in glucose metabolism. Various studies have observed substantial changes in metabolites related to glucose metabolism, including glucose and lactate, following a MI (207, 208). These changes reflect a shift in the myocardial cells' energy dependence. The measurement of these metabolites can provide valuable insights into the metabolic status of patients with MI.

Metabolomic studies have revealed a series of metabolites related to the prognosis of MI, including amino acids, lipids, and sugar metabolites. These metabolites can be used to predict adverse outcomes in patients with MI, such as major adverse cardiovascular events (MACEs), HF, death, etc. (209–211). For example, recent research has identified a significant positive correlation between heightened levels of various metabolites, such as phenylacetyl glutamine, indoxyl sulfate, deoxycholic acid, trimethylamine N-oxide, trimethyllysine, dimethylarginines, and MACEs, in individuals suffering from MI (212–214). Increased plasma kynurenine levels have been found to be positively associated with the occurrence of STEMI and its adverse outcomes (190). These findings provide valuable insights for healthcare professionals, enabling them to better assess a patient's risk and implement appropriate interventions. After a MI, the body rapidly activates its inflammatory response, resulting in increased levels of markers like interleukins and C-reactive protein, which can damage cardiac tissue (215). Metabolomic studies reveal the intricate interactions between these inflammatory molecules and metabolic compounds, providing a comprehensive understanding of inflammation's metabolic effects (216–218). Additionally, metabolic alterations have been shown to reflect energy deficit, acidosis, oxidative stress, ion imbalance, and cardiac injury following MI (191). Analyzing changes in metabolic markers allows us to assess the extent of myocardial tissue damage and predict patient prognosis.

Metabolomic studies provide valuable insights into the treatment of MI. Firstly, through metabolomics techniques, doctors can gain a better understanding of the metabolic profiles of each patient, thereby adjusting drug selection and dosages to enhance treatment effectiveness. Xia et al. (189) conducted research into the distinct metabolic changes in diabetic patients experiencing AMI. They uncovered essential metabolites linked to compromised mitochondrial function, impaired glucose utilization, and heightened inflammation. These finding sheds light on personalized therapeutic strategies for cases involving diabetes-associated AMI, representing a promising avenue for targeted interventions. Additionally, metabolomics can also contribute to the discovery of new drugs that may treat MI by intervening in metabolic pathways. Metabolomic studies have been employed to elucidate the therapeutic impacts of drugs on MI, such as the hydroethanolic extract of Cucumis sativus L. seeds (219), colchicine (220), SJP (221), and SBP (222). These studies offer a comprehensive insight into the mechanisms underlying drug treatments for MI. They hold the potential to guide the development of more effective treatments and provide valuable insights into evaluating the safety and potential side effects of drugs. Fan et al. (223) used metabolomics to reveal the therapeutic potential and synergistic mechanism of total saponins and flavonoids in notoginseng-safflower (NS-SF) in treating MI, emphasizing its superior efficacy compared to individual components and highlighting their combined regulation of key metabolic pathways in MI treatment. This study provides valuable insights for the clinical development of NS-SF as a potential treatment for CVDs. Furthermore, metabolomic studies can shed light on the impact of specific interventions in patients with MI. For instance, ketone ester supplementation has been shown to attenuate cardiac inflammation and enhance cardiac energy in a porcine model of AMI (224). Another metabolomic investigation has indicated that the combination of smoking and a high-fat diet may exacerbate cardiac dysfunction following MI, resulting in substantial disruptions in metabolic pathways related to inflammation, energy metabolism, and excessive oxidative stress (225). Therefore, gaining a deeper understanding of how different dietary patterns, supplements, and dietary modifications affect metabolic pathways could prove instrumental in enhancing the recovery and treatment outcomes of MI patients.

### 2.6. Microbiomics

Microbiomics is an interdisciplinary field that combines microbiology, macrotranscriptomics, macroproteomics, metabolomics, chemistry, culturomics, ecology, phylogeny, and systems biology to investigate the composition, diversity, and function of microorganisms (226, 227). The primary sequencing methods used in microbiomics include marker gene sequencing (e.g., 16S amplicon sequencing), macrogenome sequencing, and macrotranscriptome sequencing (228).

The human body contains over 2,000 types of microorganisms that interact in complex ways with the host (229). Disruptions in microbial ecology can promote atherosclerosis and CVD through inflammation, arterial fibrosis, and dyslipidemia (230–233). Firstly, the microbial community within the human body is closely related to the pathogenesis of MI. Research has shown that an imbalance in the gut microbiota is a key factor contributing to the development of CVDs (234). This imbalance can lead to an increase in harmful microorganisms while simultaneously decreasing the presence of beneficial microorganisms, thereby promoting an increase in inflammatory responses and disturbances in lipid metabolism. These factors may result in atherosclerosis, ultimately leading to the occurrence of MI (235). For example, short-chain fatty acids (SCFAs) are metabolites produced when intestinal microorganisms ferment dietary fibers such as cellulose. Research shows that SCFAs can have anti-inflammatory and antioxidant effects, which contribute to the maintenance of cardiovascular health (236). Nevertheless, an imbalance in gut microbes may lead to reduced SCFA production, thereby diminishing this protective effect and potentially promoting the development of MI (237). Additionally, a study has shown that the gut microbiota may impact the occurrence and development of AMI through the SCFA pathway (238). Trimethylamine oxide (TMAO) is a compound produced through intestinal microbial metabolism and is commonly associated with the consumption of fish, red meat, and other foods rich in choline and L-carnitine (239). Studies have demonstrated that elevated levels of TMAO are linked to the promotion of plaque formation, inflammatory responses, and an increased risk of cardiac events (240, 241). TMAO may also exacerbate the occurrence and progression of MI by damaging arterial endothelial function and inducing atherosclerosis. Furthermore, the gut microbiota differed significantly not only between MI patients and healthy individuals but also among subgroups of MI patients (238). Similarly, blood microbiota was also associated with MI. A study found that the structure and abundance of blood microbiota differed between MI patients and healthy individuals (242). These microbiome changes may become potential biomarkers, providing new avenues for early diagnosis and risk assessment of MI.

Microbiomics has made significant progress in MI prognosis research. In patients with STEMI, intestinal bacterial translocation was positively correlated with systemic inflammation and adverse cardiovascular events. Treatment with antibiotics to eliminate intestinal bacterial translocation alleviated systemic inflammation and myocardial cell damage in MI mice (243). Butyrate, a SCFA produced by the microbiota, can suppress inflammation and prevent myocardial hypertrophy. Research has demonstrated its capacity to enhance cardiac function and promote sympathetic nerve remodeling after MI in rats (244). Conversely, elevated plasma levels of TMAO independently correlated with a high risk of plaque rupture in STEMI patients (245). Additionally, studies have consistently indicated that high TMAO levels are positively associated with MACEs, atrial fibrillation, and coronary atherosclerotic burden (209, 213, 246–248). TMAO holds promise as a valuable biomarker for predicting poor prognosis and improving risk assessment and management in MI patients. Considering these findings, microbiomics opens up new diagnostic and therapeutic avenues for MI patients. By restoring balance to the intestinal microecology, it is anticipated to reduce chronic inflammation levels, enhance immune function, lower the risk of recurrent MI, and ultimately improve patient survival rates.

Microbiome research has made exciting advancements in MI treatment, offering innovative perspectives for novel therapeutic strategies. Firstly, research has established a strong link between the gut microbiome and chronic inflammation, a significant contributor to MI (235, 249). By modulating the intestinal microecology, it holds the potential to alleviate chronic inflammation in patients and support post-MI recovery. For instance, studies have demonstrated that supplementing with probiotics or prebiotics can restore a healthier balance of intestinal flora, effectively reducing inflammation and facilitating recovery following MI (250–252). This provides direction for the development of new biologics or dietary interventions. Secondely, studies have shown that gut microbiota remodeling through certain interventions, such as *Lactobacillus johnsonii,* dapagliflozin, and flavonoids, can result in enhanced cardiac function after AMI (253–255). SCFAs have been found to improve cardiomyocyte function and heart contractility (244, 256). Therefore, by increasing the intake of dietary fiber and promoting the production of SCFAs, it is expected to improve the cardiac function of patients with MI. Furthermore, exercise intervention can influence the gut microbiota to improve cardiac function after MI (257, 258). Thirdly, the microbiome can influence drug metabolism and effects. Studies have shown that the efficacy of some drugs, such as antibiotics (259), immunosuppressive drugs (260, 261), and anticoagulants (262), in the treatment of MI may be affected by the microbiome. Therefore, by adjusting the microbiome, drug efficacy can be optimized and therapeutic effects improved.

### 2.7. Single-cell omics

Cells are the fundamental building blocks of all living organisms, and exploring the phenotype and function of individual cells can lead to a deeper understanding of biological activities, helping to comprehend the causes, progression, and treatment of medical conditions such as MI. The use of single-cell omics has greatly advanced our understanding of cell heterogeneity, with technologies such as single-cell RNA sequencing (scRNA-seq), single-cell DNA sequencing (scDNA-seq), single-cell ATAC sequencing (scATAC-seq), and cytometry providing unprecedented levels of detail when examining individual cells (263, 264). Single-omics analysis unveils cell population diversity and functional disparities, opening a new dimension for studying diseases like MI.

Single-cell genomics has provided unprecedented insights into the responses of distinct cell types within cardiac tissue during MI. MI involves a cascade of complex biological events, including myocardial cell necrosis, infiltration of inflammatory cells, and fibroblast proliferation, among others. Traditional research methods struggle to capture the nuances and changes within these cell types. Fortunately, single-cell genomics technology empowers researchers to decode the gene expression patterns of individual cells. Through scRNA-seq of cardiac samples from MI patients, researchers have identified various cell types, including myocardial cells, immune cells, fibroblasts, and endothelial cells, and have explored their dynamic changes during MI (265–268). This comprehension of cellular diversity is pivotal in the study of cardiac development, injury responses, and the progression of cardiac diseases. In a study by Qian et al. (269), utilizing the scRNA-seq method, the transcriptomes of peripheral blood mononuclear cells in AMI patients were analyzed, revealing a total of 27 cell clusters. This investigation unveiled that peripheral immune cells in patients with plaque rupture exhibited marked pro-inflammatory characteristics, while plaque erosion was associated with intermediate monocyte expansion, neutrophil activation, and granule release. These single-cell analysis techniques enable scientists to delve deeper into the distinctive roles of various cell types in MI pathology. Research has consistently shown that myocardial cell heterogeneity is closely linked to cardiac remodeling, angiogenesis, and inflammation following MI (267, 270–274). A recent study has revealed a highly enriched regulatory T cell (Treg) subtype within the myocardium of MI mice, which facilitates cardiac damage repair post-MI (275). Furthermore, in an analysis of myocardial cells from a pig AMI model, a cell cluster potentially related to cardiac injury was identified. This cluster regulates the cardiac injury response by upregulating genes such as *TBX5*, *TBX20*, *ERBB4*, and *GRK5* (276). These research findings underscore the significance of myocardial cell heterogeneity and potential mechanisms in the treatment of MI and cardiac injury repair. Furthermore, scRNA-seq is a valuable tool for investigating communication between different types of heart cells. Understanding the network of interactions among cardiac cells that constitute the heart is crucial for comprehending cardiac homeostasis and the progression of diseases (277). Skelly et al. (278) delved into the analysis of non-muscle cells in the mouse heart using scRNA-seq technology, uncovering the diversity and sex differences among cardiac cells. This research provides essential insights for the study of cardiac development and diseases, deepening our understanding of cardiac cell composition and function. Another study conducted a focused study on the cellular composition of the mouse left ventricle. Through scRNA-seq, they identified various types of cardiac cells and elucidated their functions. This study not only offers potential therapeutic targets for CVDs but also unveils an extensive network of interconnected communication among cells within the left ventricle (279). Through a comprehensive exploration of the functions of different cell types and their interactions, we enhance our understanding of MI's pathogenesis and provide invaluable insights for future treatment strategies.

Single-cell genomics provides an opportunity to discover new biomarkers. By examining individual cells, particularly their gene expression patterns within different cell subpopulations, researchers have successfully identified a range of potential biomarkers associated with MI. For example, Zhang et al. (280) used scRNA-seq technology to reveal the potential roles that *IL1B* and *TLR2* may play in the diagnosis of MI, which are closely related to various infiltrating immune cells. Another study also identified a series of immune cell-related genes, including *FOS*, *DUSP1*, *CXCL8*, and *NFKBIA*, which can not only differentiate between AMI and coronary heart disease (CHD) but also predict the risk of HF in AMI patients (281). Furthermore, single-cell studies have revealed some monocyte-related genes, including *CUX1*, *CTSD*, *ADD3*, *PRKAR1A*, and *SDCBP*, which can be used to predict the risk of developing HF after AMI (282, 283). These findings provide new clues for early diagnosis and prognosis assessment of MI, with the potential to improve patient treatment and management. Additionally, single-cell genomics also helps uncover individual differences among MI patients. Each patient's cell composition and gene expression may vary, suggesting that personalized treatment strategies may hold promise. Single-cell research can provide a basis for developing personalized treatment plans, thereby enhancing treatment effectiveness.

### 2.8. Radiomics

Radiomics is a methodology that enables the precise characterization of pathological observations detected in radiological imaging by essentially converting images into data. Its research stages typically include data selection, medical imaging, feature extraction, exploratory analysis, and modeling (284). A The advancements in technologies such as magnetic resonance imaging (MRI), computed tomography (CT), echocardiography, nuclear imaging, and others have greatly supported accurate diagnosis and treatment in the context of MI.

MRI stands as a high-resolution non-invasive technique employed to gain intricate insights into heart structure and function, including parameters such as ventricular wall thickness, myocardial function, and volume. Recent research indicates that MRI-based radiomics, particularly in non-contrast MRI, holds great potential in predicting MI, promising to revolutionize the management and treatment of heart diseases. A study by Smith et al. (285) demonstrated the reliability of machine learning-based radiomic features extracted from non-contrast MRI in distinguishing between MI and normal tissue, offering a novel avenue for clinical diagnosis. Another study revealed that radiomic analysis using non-contrast MRI can predict adverse LVR following STEMI, thereby enhancing the accuracy of patient prognosis assessment (286). Furthermore, the integration of native T1 and extracellular volume (ECV) mapping within MRI technology, along with radiomic analysis, elevates the accuracy of predicting heart function recovery and microvascular damage. Ma et al. (287) indicated that radiomic analysis using non-contrast CMR T1 mapping can play an important role in the diagnosing AMI and the predicting myocardial function recovery. This method not only enhances the accuracy of detecting microvascular obstruction but is also expected to improve long-term predictions of myocardial contraction function, providing valuable tools for the clinical management of cardiac diseases. Additionally, radiomics based on non-contrast-enhanced T1 mapping exhibits the capacity to predict MACEs in STEMI patients, facilitating patient risk stratification (288). Chen et al. (289) unveiled that radiomic texture analysis-based ECV score mapping can differentiate between reversible and irreversible myocardial injuries in STEMI patients while predicting LVR, potentially assuming a significant role in clinical applications. Late gadolinium enhancement (LGE) is a commonly used MRI technique for detecting myocardial abnormalities. Di Noto et al. (290) found that radiomic features of LGE can accurately differentiate between MI and myocarditis, offering a promising tool for non-invasive diagnosis. Furthermore, combining LGE-based radiomics with machine learning can predict MACEs in STEMI patients (291). Therefore, MRI-based radiomics and machine learning have opened new horizons in CVD research. They can be seamlessly integrated with MRI and clinical information to enhance model accuracy and predictive performance.

CT plays a significant role in the diagnosis and evaluation of MI. Coronary CT angiography (CCTA) enables the non-invasive assessment of coronary artery narrowing and obstruction, aiding doctors in determining the patient's coronary condition. Pericardial adipose tissue (PCAT) is the fat surrounding the heart within the pericardial sac, often relevant in cardiovascular research and clinical contexts due to its potential impact on heart health (292). A recent study has discovered that the use of CCTA for the radiomic analysis of PCAT can effectively differentiate between patients with AMI and those with stable coronary arterial disease (CAD) (293). This research highlights the potential role of radiology and machine learning in cardiac disease image diagnosis. Specifically, the combination of clinical characteristics, PCAT attenuation, and radiomic parameters can enhance the accuracy of identifying AMI patients. Additionally, Si et al. (294) indicate that radiomics analysis of CCTA-based PCAT radiological features excels at distinguishing between AMI and unstable angina pectoris (UA). They found that the application of PCAT's radiomics characteristics and the fat attenuation index can improve performance in recognizing AMI, offering a promising new approach for non-invasive CHD diagnosis. Another study has also proven that radiomics features based on CCTA-derived PCAT can be used to distinguish between NSTEMI and UA. However, this study also noted certain limitations in the radiological model of epicardial adipose tissue for this task (295). Therefore, the radiomic features of PCAT based on CCTA can be used for the differential diagnosis of MI.

Therefore, radiomics plays an increasingly vital role in the diagnosis, treatment planning, and overall patient management of MI. The ongoing development and enhancement of these technologies hold the promise of delivering more precise and personalized diagnostic and treatment options for MI patients, ultimately contributing to improved recovery and survival rates. However, it's imperative to address challenges related to data privacy, standardization, and clinical integration to effectively introduce these innovations into routine clinical practice and, ultimately, to the benefit of patients.

## 3. From single-omics to multi-omics integrative analyses: toward the era of MI personalized medicine

### 3.1. Necessity of multi-omics integration in personalized medicine

As depicted in Figure 1, diverse omics exhibit unique characteristics when delving into the intricacies within organisms. The different omics provide insights into different layers of biological information. Furthermore, there exists a strong interconnectedness among these omics. Genomics concentrates on an organism's genetic information, which generally remain relatively static and stable. Conversely, other omics domains like epigenomics, transcriptomics, proteomics, and metabolomics offer dynamic insights into gene expression, protein activity, and metabolite levels (296–298). Progressing from genomics to epigenomics, and subsequently to transcriptomics, proteomics, metabolomics, and other omics, the complexity and dynamism of information progressively intensify, more effectively showcasing a wide range of disease phenotypes and contributing to the comprehension of disease origins and progression mechanisms (299). Nevertheless, as complexity deepens, data analysis becomes increasingly intricate.

Furthermore, each omics approach has its own advantages and disadvantages (Table 1). Single-omics studies offer the advantage of conducting in-depth examinations of specific biological or molecular mechanisms. There studies provide highly focused information that allows researchers to delve deeply into specific genes, proteins or molecular mechanisms, revealing microscopic level details and mechanisms. This focus makes single-omics studies particularly useful when addressing specific biological questions and research goals, such as identifying disease-causing genes, analyzing protein function, or studying a specific metabolic pathway (300). Additionally, single-omics studies usually have high experimental controllability, facilitating the acquisition of reproducible results and offering relatively low costs. This makes single-omics studies a powerful tool for studying specific biological questions or conducting preliminary explorations. However, the disadvantage of single-omics studies is their narrow focus, and they often fail to provide insights into the interactions and integration of the overall biological system. Life science often involves multi-level and complex interactions, so relying solely on data from a single research field may not fully understand the integrity and comprehensiveness of biological systems (301–303). Furthermore, each omics approach has its own limitations, including technical constraints, experimental setups, and data analysis.

**Table 1:** Advantages and disadvantages of individual omics approaches.

Omics approach	Advantages	Disadvantages
Genomics	Provides comprehensive information on the entire genome sequence, including data on all genes and non-coding regions, helping to identify important genomic changes and mutations.	Predictions of final biological effects are limited because genes do not always have a direct impact on biological performance.
Epigenomics	Provides information about potential regulatory mechanisms of genes, including DNA methylation, histone modifications, etc., thus helping to understand gene regulation and expression patterns.	Metabolite profiles may not fully capture cell type dynamics, and their correlation with gene expression can be limited due to epigenetic modifications not always directly governing gene expression levels.
Transcriptomics	Capable of comprehensive gene expression analysis, including splice variants, with high sensitivity and quantification, even in single-cell experiments for cell-specific transcriptome resolution.	Differences in organ and cell-specific transcriptomes do not always linearly translate to the protein level due to distinct regulatory mechanisms between gene and protein expression.
Proteomics	Reveals the final level of regulation within the cell, as proteins are the main cellular effectors and can directly reflect biological functions and metabolic status.	Difficulty isolating and detecting certain proteins, a high dynamic range, the need for absolute quantitative markers, limited coverage, and challenges in accurately analyzing post-translational modifications.
Metabolomics	Closely linked to phenotype while allowing repeated sampling of easily accessible biological fluids with high sensitivity and specificity	Metabolite profiles vary individually and are influenced by factors like diet, environment, and age. Research results may lack reproducibility, and there are complexities and challenges in data analysis and interpretation.
Microbiomics	Reveals the diversity and functionality of microbial communities in various environments and hosts, aiding in the understanding of the relationship between microorganisms and health.	Understanding the relationship between microbial community structure and function is often challenging, with sample collection and processing potentially distorting microbial composition.
Single-cell omics	Provides high-resolution single-cell analysis, revealing the diversity and functionality within cells while uncovering cellular heterogeneity.	Requires highly complex experimental procedures and data processing, with data analysis posing a challenge.
Radiomics	Visualizes the structure and function within the organism, offering a potent tool for pathology, anatomy, and biomedical research, thereby aiding in early disease diagnosis and treatment.	Limited image resolution challenges microstructure capture, demanding substantial resources and specialized expertise for analysis.

Therefore, a pressing need has arisen to transition from single-omics to multi-omics integrative approaches in current biomedical research. Although conventional single-omics techniques retain significance within specific fields, they encounter challenges in fully capturing the intricacies and diversity present within organisms. Particularly, when delving deeply into complex maladies like MI and the analysis of individual variabilities, single-omics approaches exhibit inherent limitations. The etiology of MI is exceptionally intricate, potentially giving rise to abnormalities across multiple levels. Genetic variations and irregularities in gene expression may hold pivotal roles in the progression of the disease. Genetic mutations could render individuals more susceptible to cardiac ailments, thereby amplifying the risk of MI (304). In parallel, aberrant gene expression could disrupt the functionality of the cardiovascular system, further hastening the onset of MI (305, 306). However, beyond genetics, subsequent alterations could serve as triggers for MI, encompassing factors such as environmental influences, lifestyles, and epigenetics (123, 307–310). This underscores that MI constitutes a multi-tiered and complex process, with deviations at each omics level potentially influencing the eventual outcome. Consequently, when confronted with the intricacies of MI, the integration of multi-omics approaches becomes pivotal, not only furnishing additional evidence but also propelling deep phenotyping analysis and the advancement of personalized medicine.

The integration of multi-omics approaches refers to the practice of combining and analyzing data from various omics technologies, such as genomics, epigenomics, transcriptomics, proteomics, and metabolomics. These approaches enable a more comprehensive understanding of biological systems, yielding profound insights into complex biological processes and diseases. By merging molecular omics with clinical phenotype omics data, we can delve into the underlying causes of MI, unravel the complex connections between genes and phenotypes, and create a detailed patient information landscape. This not only enhances the accuracy of disease diagnosis but also empowers the creation of tailor-made treatment protocols and the real-time tracking of patient responses to treatment. Thus, the shift from single-omics methods to multi-omics integrative approaches has evolved into an indispensable trend in contemporary biomedical research. This transformation paves the way for broader possibilities in future medical research and clinical practice, ushering in a new era of personalized medicine for MI.

### 3.2. Advancements in multi-omics integration research for MI

In recent years, researchers have adopted a strategy of integrating two or more “omics” approaches to conduct in-depth research on the molecular mechanisms and potential biomarkers of MI. These studies help us better understand the pathogenesis of MI and provide new clues for early diagnosis and treatment. Table 2 displays some MI research involving multi-omics integration.

**Table 2:** Studies for multi-omics integration of myocardial infarction.

Study	Year	Species	Omics types	Biomarkers or key molecules	Findings
Chen et al. (311)	2018	Human	Genomics, transcriptomics	*SIRT5*	Genetic variations significantly reduced *SIRT5* gene promoter transcriptional activity and may change *SIRT5* levels, increasing AMI risk.
Zhang et al. (312)	2018	Human	Genomics, transcriptomics	*ATG7*	Genetic variations significantly altered *ATG7* gene promoter transcriptional activity and may change *ATG7* levels, increasing AMI risk.
Sun et al. (313)	2019	Human	Genomics, transcriptomics	*GATA6*	Genetic variations (DSV g.22168409 A > G and SNP g.22168362 C > A) in the *GATA6* gene promoter may elevate *GATA6* levels, increasing AMI risk.
Wang et al. (314)	2019	Human	Genomics, transcriptomics	*VEGFR-1*	Genetic variations significantly altered *VEGFR-1* gene promoter transcriptional activity and may change *VEGFR-1* levels, increasing AMI risk.
Sedky et al. (315)	2018	Human	Genomics, metabolomics	*CYP2R1*	*CYP2R1* genetic variants strongly influenced serum 25-hydroxyl vitamin D levels and had a strong association with MI risk.
Asif et al. (316)	2018	Human	Genomics, metabolomics	*LRP8*	The TG haplotype of *LRP8* gene variants rs10788952 and rs7546246 significantly increased MI risk, along with higher low-density lipoprotein cholesterol and total cholesterol levels in MI patients.
Semaev et al. (317)	2019	Human	Genomics, metabolomics	*CETP*	The rs708272 variant in the *CETP* gene was linked to higher MI risk, correlated with reduced high-density lipoprotein cholesterol levels and increased atherogenicity.
Li et al. (17)	2020	Human	Genomics, metabolomics	*ANRIL*, *MALAT1*	Genetic variations in *ANRIL* and *MALAT1*, particularly rs9632884 and rs3200401 SNPs, were linked to lipid levels in MI patients.
Wang et al. (318)	2020	Mouse	Epigenomics, transcriptomics	H3K27ac, H3K9ac, and H3K4me3	Histone modifications regulated early MI gene expression, influencing processes like cardiomyocyte development, inflammation, angiogenesis, and metabolism, possibly through super-enhancers initiating early angiogenesis.
Corbin et al. (319)	2022	Human	Epigenomics, transcriptomics	*F2RL3*, *PAR4*	Smoking-induced DNA hypomethylation at the *F2RL3* locus may increase *PAR4* expression, potentially enhancing platelet reactivity and worsening the elevated MI risk associated with smoking.
Luo et al. (17)	2022	Mouse	Epigenomics, transcriptomics	*Ptpn6*, *Csf1r*, *Col6a1*, *Cyba*, and *Map3k14*	Substantial DNA methylation and gene expression alterations occurred in early AMI, particularly within 6 h post-AMI.
Liu et al. (320)	2022	Murine	Epigenomics, transcriptomics	*SPI1*	Increased *SPI1* expression due to reduced DNA methylation worsens MI by triggering the TLR4/NFκB pathway, resulting in heightened inflammation and cardiac damage.
Wu et al. (321)	2023	Mouse	Transcriptomics, proteomics	*Itgb2*, *Syk*, *Tlr4*, *Tlr2*, *Itgax*, *Lcp2*, *Coro1a*	Identified seven key AMI-related genes, with *Coro1a* upregulated in both omics, and strong diagnostic potential found in *Tlr2*, *Itgax*, and *Lcp2* for AMI.
Jia et al. (322)	2019	Rat	Transcriptomics, proteomics	N/A	Salvianic acid A sodium exhibited cardioprotective effects in MI by regulating multiple pathways.
Li et al. (323)	2019	Mouse	Transcriptomics, proteomics	*Nppa*, *Serpina3n*, *Anxa1*	Characterized transcriptome and proteome changes in MI, emphasizing immune, cell cycle, and ECM-related pathways, and identifying potential biomarkers for MI.
Liu et al. (324)	2023	Mouse	Transcriptomics, proteomics	WIPI1	Identified early immune activation, pyroptosis, and autophagy in myocardial tissue after AMI, and provided a potential diagnostic biomarker (WIPI1) for AMI and therapeutic implications of the pyroptosis inhibitor VX-765.
Contessotto et al. (325)	2023	Ovine	Transcriptomics, proteomics	N/A	Uncovered unique tissue remodeling traits compared to STEMI, offering insights for potential pharmacological treatments targeting fibrotic remodeling in NSTEMI.
Jia et al. (326)	2019	Rat	Proteomics, metabolomics	N/A	Salvianic acid A sodium protected against MI in rats by reversing multiple MI-induced metabolic changes and binding to specific proteins involved in metabolic pathways.
Yan et al. (327)	2022	Rat	Proteomics, metabolomics	N/A	Nutmeg-5 reduced post-MI cardiac fibrosis by inhibiting the ECM-receptor interaction pathway and TGF-β1/Smad2 signaling via plasma metabolite control.
Zhang et al. (328)	2023	Rat	Proteomics, metabolomics	Cytochrome P450 family 7 subfamily A member1 (CYP7A1)	The ethanol extract of Pueraria lobata boosted bile acid levels and alleviated gut microbiota dysbiosis in AMI by increasing CYP7A1 expression and reinstating diversity in the intestinal microbiota.
Chan et al. (329)	2020	Human, murine	Proteomics, single-cell transcriptomics	N-terminal B-type natriuretic peptide, troponin T, angiopoietin-2, thrombospondin-2, latent transforming growth factor-beta binding protein-4, and follistatin-related protein-3	Identification of potential protein biomarkers of post-MI HF and their sources.
Li et al. (330)	2023	Mouse	Proteomics, single-cell transcriptomics	Plasma exosomes	Neonatal mouse plasma exosomes (npEXO) enhanced cardiac repair and angiogenesis in adult hearts after MI through 28 npEXO ligands interacting with five cardiac endothelial cell receptors.
Zhang et al. (331)	2022	Human	Metabolomics, metagenomics	N/A	Dysbiosis of the gut microbiota significantly contributed to increased platelet reactivity in STEMI patients treated with ticagrelor after PCI.
Dong et al. (332)	2023	Human	Metabolomics, microbiomics	*Alistipes*, *Streptococcus*, *Lactobacillus*, *Faecalibacterium*, formate, methionine, tyrosine, urea, galactose	A combination of gut microbiota and fecal/urinary metabolites has yielded a set of potential, useful, and noninvasive predictive biomarkers for distinguishing AMI from stable CAD.
Liao et al. (333)	2023	Rat	Metabolomics, microbiomics	*Staphylococcusm*, *Jeotgalicoccus*, *Lachnospiraceae*, *Blautia*,eicosanoids	Suxiao Jiuxin pill's cardioprotective effects against AMI might result from its impact on gut microbiota and host fatty acid metabolism, particularly eicosanoids.
Kim et al. (334)	2023	Mouse	Single-cell transcriptomics, spatial transcriptomics	Trem2	Trem2^hi^ macrophage subsets played a role in subacute MI, displaying increased expression of anti-inflammatory genes, indicating potential therapeutic role of in Trem2 post-MI LVR.
Haase et al. (61)	2022	Mouse	Genomics, epigenomics, transcriptomics	*Gpr15*	Elevated *GPR15* expression was associated with early-onset MI, potentially mediating the adverse effects of smoking on MI risk. Additionally, DNA hypomethylation and a *GPR15* nucleotide polymorphism, rs2230344, were also linked to MI risk.
Kuppe et al. (335)	2022	Human, mouse	Epigenomics, single-cell transcriptomics, spatial transcriptomics	N/A	Created a comprehensive molecular map of human cardiac remodeling post-MI, revealing insights into cell-type shifts, transcriptome and epigenome changes, and tissue reorganization.
Lavine et al. (336)	2023	Human	Epigenomics, single-cell transcriptomics, spatial transcriptomics	*FAP*, *POSTN*, *THY-1*, *EDNRA*, *RUNX1*, *CCR2*	Discovered a subset of fibroblasts in cardiac disease, which causes tissue fibrosis. Their emergence results from interactions with *CCR2* macrophages through IL-1β signaling. Targeting inflammation to block these interactions holds potential as a therapy to reduce cardiac fibrosis and restore organ function.
Wang et al. (337)	2015	Rat	Epigenomics, transcriptomics, proteomics	*ALDH2*	Abnormal hypermethylation of CpG sites in the *ALDH2* promoter upstream sequence was linked to myocardial ischemic injury and contributes to *ALDH2* mRNA and protein downregulation after MI.
Lan et al. (338)	2022	Mouse	Epigenomics, transcriptomics, proteomics	*DYRK1A*	Inhibiting *DYRK1A* activated the cardiomyocyte cell cycle and enhanced cardiac repair after MI, with a crucial role played by epigenetic modifications (H3K4me3 and H3K27ac) involving WD repeat-containing protein 82 and lysine acetyltransferase 6A.
Ward-Caviness et al. (339)	2018	Human	Epigenomics, transcriptomics, metabolomics	*KCNN1*, *FRY*, *LRP8*, *DHCR24*, *GLIPR1L2*, *ALKBH1*, *PDE4DIP*, *C1orf129*	Significant changes in DNA methylation occur after MI and were associated with alterations in branched-chain amino acid metabolism.
Hadas et al. (340)	2020	Mouse	Transcriptomics, proteomics, metabolomics	Acid ceramidase	Elevated acid ceramidase via modified mRNA delivery lowers ceramide levels, boosts cell survival, and offers cardioprotection post-MI by enhancing heart function and reducing scar size.
Jiang et al. (93)	2020	Mouse	Transcriptomics, proteomics, metabolomics	*HDAC4, GLUT1*	Exercise enhances cardiac function and glucose metabolism in HF mice post-MI induced by ischemia by inhibiting *HDAC4* and increasing *GLUT1* expression via AMPK-HDAC4-MEF2a pathway activation.
Lim et al. (341)	2022	Human	Metabolomics, lipidomics, glycomics, metallomics	N/A	Unraveled the potential mechanisms of glycerophospholipids, Ca-ATPases, and phosphatidylethanolamine. The combination of all four omics datasets significantly enhanced AMI classification.

AMI, acute myocardial infarction; MI, myocardial infarction; DSV, dynamic structured variant; SNP, single nucleotide polymorphism; ECM, extracellular matrix; WIPI1, WD repeat domain, phosphoinositide interacting 1; HF, heart failure; PCI, percutaneous coronary intervention; CAD, coronary artery disease; Trem2, triggering receptor expressed on myeloid cells 2.

Multi-omics integration analysis is a powerful tool for exploring the relationships between molecules at various levels in MI and identifying potential biomarkers and key molecules. The integrated research of genomics and transcriptomics is used to study the relationship between genetic variations and changes in transcriptional expression, which is conducive to clarifying the potential molecular mechanisms of MI. Chen et al. (311) revealed that genetic variations significantly reduce the transcriptional activity of the *SIRT5* gene promoter. This alteration could result in changes in *SIRT5* levels, thereby increasing the risk of AMI. *SIRT5* overexpression has been demonstrated to provide protection against cardiac dysfunction induced by pressure overload, while simultaneously suppressing adverse metabolic and fibrotic pathways associated with HF (342). Similarly, other studies have uncovered that genetic variations in *ATG7* (312), *GATA6* (313), and *VEGFR-1* (314) can significantly impact the transcriptional activity of these genes, further influencing the risk of MI. Furthermore, Haase et al. (61) employed a combination of genomics, epigenomics, and transcriptomics to reveal that elevated *GPR15* expression is associated with early-onset MI, potentially mediating the adverse effects of smoking on MI risk. Additionally, they found that DNA hypomethylation and a nucleotide polymorphism (rs2230344) of *GPR15* are also linked to MI risk. Since rs2230344 is located in close proximity to *GPR15* DNA methylation sites (343), it may influence DNA methylation, leading to increased GPR15 levels. The integrated research of genomics and metabolomics is employed to explore the intricate connections between genetic variations and alterations in metabolite profiles. 25-hydroxy vitamin D (25OHD) serves as an intermediary product in the body's vitamin D metabolism, participating in lipid metabolism. Its deficiency may increase the risk of MI (344). Sedky et al. (315) revealed that genetic variations in the *CYP2R1* gene can modulate serum 25OHD levels, thereby impacting MI risk. Lipid metabolism abnormalities are intricately linked to the risk of MI, as elevated cholesterol levels and imbalanced lipid profiles can contribute to atherosclerosis and heighten the likelihood of MI (345). Studies have indicated that genetic variations in the *LRP8* (rs10788952 and rs7546246) (316), *CETP* (rs708272) (317), *NRIL* (rs9632884) and *MALAT1* (rs320040) (17) are closely associated with MI risk by exerting their influence on lipid metabolism. These research findings underscore the potential significance of genetic variations in the pathogenesis of MI, offering promising avenues for future disease treatment and personalized medicine.

The integrated research in epigenomics and transcriptomics is utilized to investigate the impact of epigenomic alterations on gene expression and to identify key molecular signatures and potential mechanism. A recent study has revealed that substantial alterations in DNA methylation and gene expression take place in the early phases of AMI, particularly within the initial 6 h following AMI. Additionally, the study has identified promising epigenetic-based biomarkers for early clinical diagnosis and potential therapeutic targets for AMI, which include *Ptpn6*, *Csf1r*, *Col6a1*, *Cyba*, and *Map3k14* (305). Wang et al. (318) unveiled the critical roles of histone modifications such as histone H3 lysine 27 acetylation (H3K27ac), histone H3 lysine 9 acetylation (H3K9ac), and H3K4me3 in the early stages of MI. They identified that at least 195 genes were upregulated and associated with one of these modifications, impacting various key biological processes, including cardiac cell development, inflammation, vascularization, and metabolism. Additionally, they discovered that enhancers rich in H3K27ac may play a crucial role in early vascular responses. In another study, researchers discovered that knocking down *DYRK1A* enhanced the expression of numerous genes involved in cell proliferation, thereby activating the cell cycle of cardiac cells and promoting cardiac repair following MI. Throughout this process, epigenetic modifications, particularly H3K4me3 and H3K27ac modifications, played a crucial role, involving proteins such as WD repeat-containing protein 82 and lysine acetyltransferase 6A. Therefore, by regulating these epigenetic modifications and protein activity, it may be possible to promote more effective cardiac cell repair, potentially leading to improved recovery and cardiac function in MI patients (338). Spleen focus forming virus proviral integration oncogene (SPI1) belongs to the ETS family of transcription factors and participates in a wide range of cellular processes, including inflammation and cell apoptosis (346, 347). Liu et al. (320) revealed that the upregulation of *SPI1* gene expression, driven by reduced DNA methylation, leads to heightened inflammation and cardiac damage through the activation of the TLR4/NFκB pathway, further exacerbating MI development. This research sheds light on the pivotal role of *SPI1* in the pathological process of MI and establishes a foundation for exploring new intervention strategies. Aldehyde dehydrogenase 2 (ALDH2) downregulation is related to MI severity, and epigenetic changes may contribute to its downregulation (348). Researchers found that *ALDH2* mRNA and protein downregulation after MI was partly due to CpG hypermethylation in the upstream *ALDH2* gene promoter, as revealed by multi-omics analysis in the rat MI model (337). The integration of epigenomics and transcriptomics provides important insights into the mechanisms linking smoking and MI. A recent study discovered that smoking can induce DNA demethylation at the *F2RL3* site, potentially increasing the expression level of the *PAR4* gene. This alteration may lead to heightened platelet reactivity, thereby escalating the risk of MI in smokers (319). Furthermore, Ward-Caviness et al. (339) correlated epigenetic fingerprint sites with cis-gene expression and integrated them into the gene expression metabolomics network. They found that DNA methylation changes in MI were associated with alterations in branched-chain amino acid metabolism. This discovery suggests that epigenetic changes may play a vital role in metabolic regulation after MI. This has significant implications for our understanding of the metabolic regulation of MI and for identifying potential therapeutic pathways. Collectively, these studies underscore the regulatory role of epigenomics in the pathogenesis of MI and provide valuable insights and targets for understanding and treating this condition.

Liu et al. (324) conducted an analysis of transcriptomes and proteomes in myocardial tissue at various time points following AMI. They noted that the earliest significant changes occurred at 6 h after AMI, and pyroptosis was activated at 24 h after AMI. Additionally, they highlighted the potential of the pyroptosis inhibitor VX-765 as a promising drug target and identified the protein WIPI1 (WD repeat domain, phosphoinositide interacting 1) as a valuable early diagnostic biomarker for AMI. Another study also revealed transcriptomic and proteomic changes associated with MI, with a particular emphasis on changes in immune, cell cycle, and ECM-related pathways. In addition, the study identified potential blood markers that could be used for the diagnosis or treatment monitoring of MI. Wu et al. (321) employed an integrated approach combining transcriptomics and proteomics to successfully identify seven key genes associated with AMI, with *Coro1a* exhibiting upregulation in both omics levels. This pivotal discovery not only presents potential new biomarkers for early AMI diagnosis but also offers hope for enhanced treatment and patient management strategies. Notably, the *Coro1a* gene, a member of the Coronin family, primarily functions within the immune system, and its aberrations can lead to immune system dysfunction and other health complications (349). Furthermore, a recent study that integrated transcriptomics and proteomics has unveiled tissue remodeling signatures specific to NSTEMI. In conjunction with the elevation of inflammation and fibrosis markers, the ischemic regions of NSTEMI displayed unique patterns of complex galactosylated and sialylated N-glycans within cellular membranes and the ECM (325). These findings offer valuable insights into potential drug therapies aimed at addressing fibrous remodeling in NSTEMI, presenting novel perspectives for future therapeutic strategies.

Integrated analysis of metabolomics and other omics has been used to uncover the protective mechanisms of drugs against MI. Sodium salvianolic acid A (SAAS) is a novel drug derived from a traditional Chinese medicine Salvia miltiorrhiza. It is currently undergoing Phase I clinical trials in China for the treatment of CHD and stable angina. Through proteomics and metabolomics analyses, Jia et al. (326) revealed that SASS possesses the remarkable ability to counteract a multitude of metabolic alterations induced by MI. SAAS exerts this protective effect by selectively binding to specific proteins integral to metabolic pathways, thereby effectively safeguarding rats from the detrimental consequences of MI. Furthermore, Jia et al. (322) utilized transcriptomic and proteomic analyses to reveal the cardioprotective effects of SAAS in MI by regulating pathways such as the actin cytoskeleton, phagosomes, focal adhesions, and others. These studies offer valuable insights into SAAS treatment for MI and pave the way for innovative research directions in future treatment strategies. Nutmeg-5 is an ancient and classic formula in traditional Mongolian medicine composed of five traditional Chinese medicines, widely used in the treatment of MI. Yan et al. (327) further elucidated the mechanisms underlying Nutmeg-5's protective efficacy against MI by employing proteomics and metabolomics analyses. Their findings revealed that Nutmeg-5 achieves its protective effect on MI through the inhibition of ECM-receptor interaction pathways and TGF-β1/Smad2 signaling transduction, which was achieved by regulating plasma metabolites. Acid ceramidase (AC) serves as the primary enzyme responsible for catalyzing the hydrolysis of ceramide, thereby producing free fatty acids and sphingosine. Recent research has highlighted the promising potential of AC gene therapy in alleviating pulmonary arterial hypertension with right heart dysfunction (350). In a study by Hadas et al. (340), utilizing a comprehensive approach encompassing transcriptomics, proteomics, and metabolomics, it was revealed that overexpressing AC via modified mRNA (modRNA) delivery can lower ceramide levels, promote cell survival, and offer cardiac protection after MI by improving heart function and reducing scar size. This discovery underscores the therapeutic potential of AC modRNA in ischemic heart disease. Zhang et al. (331) utilized metabolomics and metagenomics analyses to reveal that gut microbiota dysbiosis is a key contributing factor to high platelet activity in STEMI patients receiving ticagrelor treatment after PCI. This finding underscores the importance of gut microbiota in treating MI patients and offers new potential strategies for improving platelet activity. Liao et al. (333) used metabolomics and microbiomics analyses and discovered that the cardiac protective effects of SJP in AMI may be linked to its impact on gut microbiota and host fatty acid metabolism, especially its regulation of eicosanoids. This study offers valuable insights into the mechanism of this traditional Chinese medicine formulation and provides new avenues for AMI treatment and prevention. Furthermore, multi-omics analysis can also be used to reveal the protective effect of exercise on MI. Jiang et al. (93) conducted an integrated analysis of transcriptomics, proteomics, and metabolomics, shedding light on the beneficial effects of exercise in rats suffering from HF. Their investigation revealed that exercise not only enhances heart function but also improves glucose metabolism in HF rats through the activation of the AMPK-HDAC4-MEF2a pathway, inhibition of *HDAC4* activity, and enhancement of *GLUT1* expression. These discoveries provide novel and valuable insights into the potential treatment of HF following MI.

With the advancement of single-cell and multi-omics technologies, single-cell analysis has entered the multi-omics era. Multi-omics single-cell analysis integrates multiple types of omics information, such as genomics, epigenomics, transcriptomics, proteomics, metabolomics, or spatial state, simultaneously at single-cell resolution. This provides a more comprehensive understanding of cellular states and fates, which is of great significance for the development of precision medicine for MI. Kuppe et al. (335) developed a multi-omics map of MI using single-cell gene expression, chromatin accessibility, and spatial transcriptomic profiling. They created a comprehensive molecular map of human cardiac remodeling post-MI, revealing insights into cell-type shifts, transcriptome and epigenome changes, and tissue reorganization. These findings have significant implications for understanding cardiac diseases and potential therapeutic approaches. Kim et al. (334), through the use of single-cell transcriptomics and spatial transcriptomics techniques, have unveiled the heterogeneity of macrophages in the post-MI heart and identifies Trem2^hi^ macrophages as potential therapeutic targets. Additionally, they found that the injection of soluble form of triggering receptor expressed on myeloid cells 2 (Trem2) during the subacute phase of MI improved myocardial function and remodeling, offering promise for novel MI treatments. In another study, a comprehensive approach involving multi-omic single-cell gene expression analysis, epitope mapping, and chromatin accessibility profiling identified a specific subset of fibroblasts in human cardiac disease that contributes to tissue fibrosis. This study found that their emergence is driven by interactions with C-C chemokine receptor type 2 (CCR2) macrophages via interleukin 1 beta (IL-1β) signaling. These discoveries emphasize the broader therapeutic potential of targeting inflammation as a strategy to combat tissue fibrosis and restore normal organ function (336).

Furthermore, multi-omics data integration analysis enables the development of biomarkers for early diagnosis and personalized medicine of MI. Lim et al. (341) conducted a comprehensive investigation utilizing metabolomics, lipidomics, glycomics, and metallomics approaches to construct intricate multi-omics maps of interconnected biomolecules, significantly advancing our comprehension of MI. Their research not only unveiled the potential role of glycerophospholipids in immune regulation mediated by N-glycans but also underscored the critical importance of sarcoplasmic reticulum Ca-ATPase (SRCA) in CVD. Additionally, they elucidated the contribution of phosphatidylethanolamines to SRCA function. These findings provide pivotal insights into the molecular mechanisms of MI. Furthermore, their multi-omics classifier exhibited exceptional performance in distinguishing AMI cases from healthy subjects, achieving an impressive AUC (Area Under the Curve) of 0.953. Dong et al. (332) employed an integrated approach, combining metabolomics and metagenomics analyses, and successfully identified four gut microbiota species (*Alistipes*, *Streptococcus*, *Lactobacillus*, *Faecalibacterium*), three critical fecal metabolites (formic acid, threonine, tyrosine), and two urinary metabolites (urea, lactulose) associated with MI. The combination of these factors yielded a set of potential, valuable, and non-invasive biomarkers for distinguishing AMI from stable CAD, with an AUC of 0.932. These findings provide a valuable predictive tool with significant clinical applications. Chan et al. (329) used proteomics and single-cell transcriptomics methods to identify several potential protein biomarkers for diagnosing HF in patients following MI. These biomarkers, including N-terminal B-type natriuretic peptide, cTnT, angiopoietin-2, thrombospondin-2, latent transforming growth factor-beta binding protein-4, and follistatin-related protein-3, hold promise for early HF diagnosis and treatment. Furthermore, the study elucidated the sources of these biomarkers, with *NPPB* and *TNNT2* displaying the highest gene expression levels in cardiac muscle cells. In summary, integrating and analyzing multi-omics data holds significant promise in advancing early diagnosis and personalized medicine for MI. By analyzing multi-omics data, we can gain a more comprehensive understanding of this severe CVD, identify potential biomarkers and treatment targets, and thereby provide more accurate diagnoses, prognosis assessments, and personalized treatment plans.

## 4. Databases and online tools for multi-omics integrative analyses

### 4.1. Databases and knowledge bases

Numerous omics databases and knowledge bases offer researchers invaluable resources to integrate and analyze diverse biological data across various molecular levels, encompassing genomics, transcriptomics, proteomics, metabolomics, and more. These resources facilitate in-depth exploration of interactions between genes, proteins, metabolites, and offer comprehensive insights into biological pathways, disease mechanisms, and beyond. Table 3 presents a series of databases and knowledge bases available for integrating multi-omics data of MI. Among these, the CVD Knowledge Portal (CVDKP) (351), MI Knowledge Base (MIKB) (10), and HeartBioPortal (352) concentrate on multi-omics data pertaining to CVDs and MI, providing robust support for cardiovascular research. The Genome-Wide Association Study (GWAS) Catalog standardizes genomic association study data, streamlining analyses of genetic variations linked to traits (353). ArrayExpress serves as a repository for gene expression data, furnishing rich resources for the study of gene regulation and expression patterns (354). The GTEx project (355) directs attention toward tissue-specific gene expression, offering precious resources for comprehending gene functions in distinct tissues. Furthermore, the European Nucleotide Archive (ENA) (356) functions as a repository for nucleic acid sequencing data, playing a critical role in genomics and transcriptomics research by furnishing extensive datasets.

**Table 3:** Databases for multi-omics integrative analysis.

Database	Description	Omics types	Website	PMID
CVD Knowledge Portal (CVDKP)	It offers access to human genetic data and epigenomic annotations associated with myocardial infarction (MI), atrial fibrillation, and related traits.	Genomics, epigenomics	https://cvd.hugeamp.org/	– (351)
MI Knowledge Base (MIKB)	It is an open-access, curated database integrating multi-omics knowledge about MI to enhance translational research and provide comprehensive insights into its pathogenesis and risk factors.	Diverse omics data	http://www.sysbio.org.cn/mikb/	34900127 (10)
HeartBioPortal	It integrates publicly available gene expression data and genetic association content to harness the power of transcriptomics in revealing the effects of genetic variation on gene expression and alternative splicing in health and disease.	Genomics, transcriptomics	https://heartbioportal.com	31294639 (352)
Genome-Wide Association Study (GWAS) Catalog	It provides standardized and detailed GWAS data, supporting diverse research communities with variant-trait associations, summary statistics, and expanded data types.	Genomics	https://www.ebi.ac.uk/gwas	36350656 (353)
ArrayExpress	It is a publicly accessible database that provides a comprehensive repository of high-quality functional genomics data, including gene expression and molecular profiling information.	Transcriptomics	https://www.ebi.ac.uk/biostudies/arrayexpress	30357387 (354)
Genotype-Tissue Expression (GTEx) Project	It facilitates the study of tissue-specific gene expression and regulation through an extensive collection of samples and open-access data on gene expression, quantitative trait locus (QTLs), and histology images.	Genomics, transcriptomics	https://gtexportal.org/home/	23715323 (355)
European Nucleotide Archive (ENA)	It stores a comprehensive record of the world's nucleotide sequencing information, including raw data, sequence assembly information, and functional annotation.	Genomics, transcriptomics, metagenomics	https://www.ebi.ac.uk/ena/browser/home	36399492 (356)
ProteomeXchange consortium	It is a collaborative effort among six members, including PRIDE and others, aiming to standardize mass spectrometry proteomics data submission, sharing, and dissemination for improved data management and re-use.	Proteomics	http://www.proteomexchange.org/	36370099 (357)
PeptideAtlas repository	It provides a comprehensive platform for the storage, sharing, and exploration of peptide and protein mass spectrometry data.	Proteomics	http://www.peptideatlas.org	16381952 (358)
Proteomics Identifications Database (PRIDE)	It serves as a public repository aimed at storing, sharing, and analyzing mass spectrometry-based proteomics datasets.	Proteomics	https://www.ebi.ac.uk/pride/archive/	23203882 (359)
Research Collaboratory for Structural Bioinformatics Protein Data Bank (RCSB PDB)	It a renowned initiative dedicated to collecting, curating, and disseminating valuable 3D structural information about biological macromolecules to enable advanced research in the field of molecular biology and bioinformatics.	Proteomics	https://www.rcsb.org/	36420884 (360)
ProteomicsDB	It provides researchers with enhanced data accessibility, advanced visualizations, and integration with predictive tools, thereby facilitating impactful discoveries in multi-omics research.	Proteomics, transcriptomics, phenomics	https://www.ProteomicsDB.org	34791421 (361)
Human Protein Atlas	It provides a map of all the human proteins in cells, tissues, and organs using integration of various omics technologies, including imaging, genomics, transcriptomics, proteomics, metabolomics, and functional data.	Genomics, transcriptomics, proteomics, metabolomics, imaging	https://www.proteinatlas.org/	21139605 (362)
Metabolomics WorkBench	It is a publicly accessible repository offering metabolomics metadata, experimental data, standards, protocols, metabolite structures and other resources across various species and experimental platforms.	Metabolomics	http://www.metabolomicsworkbench.org/	26467476 (363)
MetaboLights	It houses raw experimental data, associated metadata, metabolite structures, and reference spectra.	Metabolomics	http://www.ebi.ac.uk/metabolights/	31691833 (364)
Global Natural Products Social Molecular Networking (GNPS)	It is an online platform that facilitates the sharing and analysis of mass spectrometry data related to natural products.	Mass spectrometry data	http://gnps.ucsd.edu	27504778 (365)
Human Cell Atlas	It is a global collaborative initiative aimed at creating a comprehensive and detailed map of all cell types and states in the human body to advance our understanding of health and disease.	Single-cell omics	https://www.humancellatlas.org/	29206104 (366)
Single Cell Portal (SCP)	It is a platform for sharing, visualizing, and exploring single-cell omics data, enhancing research in the single-cell field.	Single-cell omics	https://singlecell.broadinstitute.org/single_cell	37502904 (367)
Database of Deeply Integrated Single-Cell Omics data (DISCO)	It offers researchers an accessible and powerful platform to explore, integrate, and analyze single-cell omics data.	Single-cell omics	https://www.immunesinglecell.org/	34791375 (368)
National Center for Biotechnology Information (NCBI)—multiple databases	It is a comprehensive resource providing access to a vast array of biomedical and genomic information, databases, tools, and services to support research, analysis, and understanding of biological data.	Genomics, epigenomics, transcriptomics, proteomics	https://www.ncbi.nlm.nih.gov/	36370100 (369)
European Molecular Biology Laboratory European Bioinformatics Institute (EMBL-EBI)	It is a world-leading research institution that specializes in collecting, curating, and providing access to a vast array of biological data and resources to support global scientific endeavors in the field of molecular biology and bioinformatics.	Genomics, transcriptomics, proteomics, metabolomics	https://www.ebi.ac.uk/	36477213 (370)
Search Tool for the Retrieval of Interaction Gene/Proteins (STRING)	It is an essential resource that gathers, assesses, and integrates protein-protein interactions, playing a vital role in enhancing our understanding of cellular functions and relationships.	Genomics, transcriptomics, proteomics	https://string-db.org/	36370105 (371)
Reactome	It is a powerful and evolving knowledgebase that provides detailed insights into cellular processes, disease annotations, and functional relationships, contributing significantly to advancing our understanding of biological complexity.	Genomics, transcriptomics, proteomics, metabolomics, glycomics	https://reactome.org	34788843 (372)
Kyoto Encyclopedia of Genes and Genomes (KEGG)	It is a powerful bioinformatics resource that offers valuable insights into genes, pathways, diseases, and drugs, playing a crucial role in advancing our understanding of biological processes and their applications in diverse scientific disciplines.	Genomics, transcriptomics, proteomics, metabolomics, pharmacomics	https://www.kegg.jp	36300620 (373)

ProteomeXchange consortium (357), PeptideAtlas repository (358), Proteomics Identifications Database (PRIDE) (359), Research Collaboratory for Structural Bioinformatics Protein Data Bank (RCSB PDB) (360), and Human Protein Atlas (362) supply ample proteomics and MS data, aiding researchers in probing protein structure and function. Human Cell Atlas (366), Single Cell Portal (SCP) (367), and Database of Deeply Integrated Single-Cell Omics data (DISCO) (368) zero in on single-cell genomics, providing pivotal platforms to uncover cellular heterogeneity and dynamic changes. Metabolomics WorkBench (363), MetaboLights (364), and Global Natural Products Social Molecular Networking (GNPS) (365) concentrate on exploring metabolomics data, underpinning research on metabolic pathways and bioactive molecules.

The National Center for Biotechnology Information (NCBI) (369) and the European Molecular Biology Laboratory European Bioinformatics Institute (EMBL-EBI) (370) stand as pivotal bioinformatics resource centers, pivotal in integrating, storing, and disseminating biological data, tools, and knowledge, thereby supporting interdisciplinary multi-omics analyses and in-depth exploration within the global life science research community. Search Tool for the Retrieval of Interaction Gene/Proteins (STRING) (371), Reactome (372), and Kyoto Encyclopedia of Genes and Genomes (KEGG) (373) provide researchers with the means to delve deeply into protein interactions, pathways, and functional annotations, furnishing essential insights for investigating molecular mechanisms. The presence of these databases lends strong support to interdisciplinary research, propelling the cross-application and analysis of multi-omics data, yielding invaluable insights for life science research, and further advancing our comprehension of the intricate nature of biological systems.

### 4.2. Analysis tools

Integrative analysis of multi-omics data is a complex and vital task, involving combining information from different omics layers to uncover deeper biological insights. Researchers can use various tools, categorized into three main types: toolkits, desktop applications, and web-based tools. Toolkits are designed for skilled programmers and typically require installation and setup. They offer extensive functions and flexibility, allowing researchers to customize analysis workflows and settings. Common toolkits include Bioconductor (R language) (374), mixOmics (R language) (375), Seurat (R language) (376), scikit-learn (Python) (377), and scGREAT (Python) (378). However, using toolkits requires programming and data skills, making it challenging for non-experts. Users also need their own computing and storage resources. Desktop applications provide graphical interfaces, enabling non-programmers to conduct multi-omics analysis. For example, Taverna (379) and KNIME (380) offer graphical user interface-based workflow design for integrating diverse omics data. However, desktop apps might have limitations in analysis due to computing and data constraints.

Web-based online tools provide a convenient solution for conducting integrative analysis of multi-omics data. These tools don't require installation and can be accessed directly through web browsers. They typically feature user-friendly interfaces that guide users step-by-step through the analysis process using graphical interfaces, eliminating the need for programming skills. However, these online tools might have limitations due to server computational resources and data privacy concerns, potentially restricting their usability for large-scale data analysis. Users could encounter constraints related to computational resources, affecting the speed of analysis. Furthermore, as the analysis workflows are predefined, customization options for analyses could be limited. Table 4 shows several integrative multi-omics online tools along with their descriptions. For example, LDexpress (381) specializes in integrating population-specific linkage disequilibrium data and tissue-specific gene expression information to investigate the impact of germ cell lineage variations on gene expression and disease associations. Paitomics (382) integrates transcriptomics and metabolomics data, Quickomics (383) combines transcriptomics and proteomics data, and GeneTrail (384) encompasses genomics, transcriptomics, miRNAomics, proteomics, epigenetics, and single-cell data integration. TIMEOR (385) integrates genomics, transcriptomics, and proteomics data to unveil temporal changes in gene regulatory networks. Omics Integrator (386) uses network optimization algorithms to integrate proteomics, gene expression, and epigenetics data. Mergeomics (387) is used to combine various types of omics data to uncover disease pathways and potential drugs. These tools offer researchers a wide range of choices to explore and interpret multi-omics data, leading to profound insights into biological processes more easily. However, when choosing an appropriate tool, users should carefully consider factors like data scale, available computational resources, privacy requirements, and analysis objectives.

**Table 4:** Online tools for multi-omics integrative analysis.

Tool	Description	Omics types	Website	PMID
LDexpress	It is an online tool that integrates population-specific linkage disequilibrium data and tissue-specific gene expression information to explore the impact of germline variation on nearby gene expression and disease associations.	Single nucleotide polymorphism, gene expression	https://ldlink.nih.gov/?tab=ldexpress	34930111 (381)
Paintomics	It is a web server for multi-omics data analysis, offering improved pathway database support, enhanced metabolite analysis, and a regulatory omics module for comprehensive insights.	Transcriptomics, metabolomics	http://www.paintomics.org	35609982 (382)
Quickomics	It empowers biologists with user-friendly modules, customizable options, and publication-ready visualizations for exploring and analyzing omics data with ease.	Transcriptomics, proteomics	http://quickomics.bxgenomics.com	33901288 (383)
GeneTrail	It facilitates integrated analysis of various molecular datasets, including transcriptomic, miRNomic, genomic, and proteomic data, offering diverse statistical tests, reference sets, and biological categories while enabling direct result comparisons.	Genomics, transcriptomics, miRNomic, proteomics, epigenetics, single-cell omics	https://genetrail2.bioinf.uni-sb.de	32379325 (384)
TIMEOR	It leverages ordered multi-omics data to reveal gene regulatory networks and mechanisms over time, offering researchers a valuable avenue for gaining insights into complex biological processes.	Genomics, transcriptomics, proteomics	http://timeor.brown.edu	34125906 (385)
Omics Integrator	It is a powerful tool that utilizes network optimization algorithms to integrate multiple omic data types, revealing underlying molecular pathways and enabling the discovery of unannotated connections.	Proteomics, gene expression, epigenetics	http://fraenkel-nsf.csbi.mit.edu/omicsintegrator/	27096930 (386)
Mergeomics	It is a free online tool for integrating multi-omics data to reveal disease pathways, networks, key drivers, and potential drugs.	Genomics, epigenomics, transcriptomics, proteomics, metabolomics	http://mergeomics.research.idre.ucla.edu	34048577 (387)
Online Resource for Integrative Omics (ORIO)	It is a web-based platform that enables intuitive and versatile analysis and integration of next-generation sequencing (NGS) data from various sources and techniques.	Genomics, epigenomics, transcriptomics, proteomics, metabolomics	https://orio.niehs.nih.gov/	28402545 (388)
GraphOmics	It is a user-friendly platform that integrates and explores multiple omics datasets by connecting biological entities based on biochemical relationships and mapping them to pathways.	Transcriptomics, proteomics, metabolomics	https://graphomics.glasgowcompbio.org/	34922446 (389)
3Omics	It is a one-click web tool that streamlines the integration and analysis of transcriptomics, proteomics, and metabolomics data, providing visualizations and insights into relationships, functions, pathways, and enrichments across multiple omics datasets	Transcriptomics, proteomics, metabolomics	https://3omics.cmdm.tw/	23875761 (390)
MOVIS	It empowers researchers to efficiently explore time-series multi-omics data, creating valuable insights and reproducible visualizations with simplicity.	Diverse omics data	https://movis.mathematik.uni-marburg.de/	35284047 (391)
xMWAS	It is a versatile software that integrates, visualizes, clusters, and analyzes diverse omics data from multiple platforms, enabling the identification of sub-networks and topological changes in systems biology studies.	Diverse omics data	https://kuppal.shinyapps.io/xmwas/	29069296 (392)
OmicsNet	It enables researchers to visualize, analyze, and gain valuable insights from multi-omics data, fostering deeper understanding of complex biological systems.	Diverse omics data	www.omicsnet.ca	35639733 (393)
MicrobioSee	It offers researchers an intuitive and efficient toolkit for visualizing complex multi-omics data and simplifying analysis.	Diverse omics data	https://microbiosee.gxu.edu.cn	35464838 (394)
Visual Omics	It offers a seamless and intuitive solution for omics data analysis and visualization, enabling researchers to effortlessly generate customized and publication-ready charts that effectively communicate their findings.	Diverse omics data	http://bioinfo.ihb.ac.cn/visomics	36458930 (395)
Interactive Data Explorer (OmicsTIDE)	It is a versatile bioinformatics tool designed to seamlessly integrate diverse numerical omics datasets, through concatenation and k-means clustering, enabling comprehensive multi-omics analysis and visualization of clustered associations.	Diverse omics data	http://omicstide-tuevis.cs.uni-tuebingen.de/	36698763 (396)
Galaxy	An open, web-based platform for data intensive biomedical research, which allows users to perform, reproduce, and share complete analyses.	Diverse omics data	https://usegalaxy.org/	35446428 (397)

## 5. Conclusion and perspectives

MI is a complex CVD, and its occurrence and development are associated with pathological changes in multiple biological systems. Single-omic approaches, such as genomics, epigenomics, transcriptomics, proteomics, and metabolomics, have greatly advanced our understanding of the molecular mechanisms of MI. However, single-omics approaches possess limitations, as they fail to fully account for the complex interactions among molecules across various levels within the context of the disease. By integrating data from various omics approaches, multi-omics research goes beyond the limitations of single omics methods and addresses gaps in information, revealing a comprehensive understanding of the causes of MI from different perspectives. This approach helps us better understand the complete range of molecular changes in MI, which in turn assists in identifying important biomarkers for diagnosis and treatment. Importantly, multi-omics integration is not only effective in revealing disease mechanisms but also has significant potential in the field of drug therapy. Analyzing data across different omics layers allows us to gain deeper insights into how drugs work in treating MI. This aids in developing more personalized and precise treatment strategies, optimizing treatment effectiveness, and minimizing the risk of adverse reactions. As a result, multi-omics integration not only helps uncover the complex molecular mechanisms of MI but also strongly supports the advancement of precision medicine and individualized treatment approaches. This approach offers new perspectives and possibilities for better understanding, prevention, and treatment of this serious condition.

**Figure 2:**
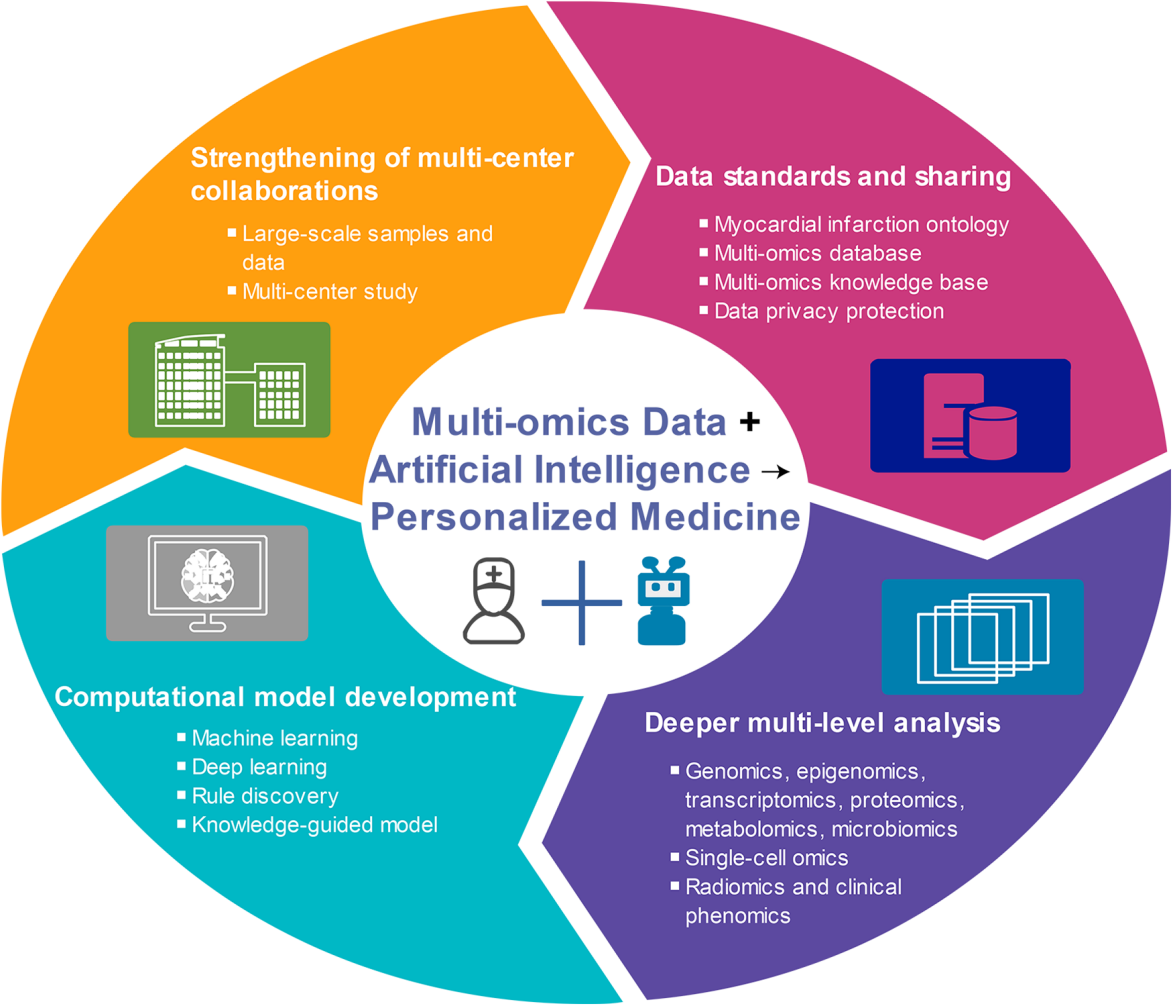
Prospective directions in multi-omics research for myocardial infarction.

Currently, multi-omics studies on MI are predominantly focused on individual omics layers, lacking comprehensive integration This limitation hinders the realization of personalized medicine in MI. Furthermore, the diversity of data and the lack of standardization make cross-study comparisons and integrated analyses challenging. Simultaneously, the high-dimensionality of multi-omics data requires more advanced algorithms and models to uncover underlying correlations. To advance precise medical research on MI, future directions in multi-omics studies should emphasize the following aspects, as illustrated in Figure 2: Firstly, conducting large-scale, multicenter, standardized clinical studies is essential to gather extensive and diverse multi-omics data on MI. This approach enhances statistical power, robustness, and replicability of research outcomes. Secondly, the establishment of unified data standards and sharing platforms is crucial. This includes constructing a MI ontology, databases, and knowledge repositories. Such practices ensure data quality and consistency, enhancing comparability across different studies. Sharing platforms foster collaboration and information exchange among researchers, driving continuous progress in multi-omics studies of MI (398, 399). Thirdly, future research should delve deeper into multi-omics analyses. Beyond genomics, transcriptomics, proteomics, and metabolomics, other omics layers such as epigenomics, microbiomics, and clinical phenomics could be explored. Integrating data from these diverse layers can provide comprehensive information, offering stronger support for accurate MI diagnosis and individualized treatment. Fourthly, the development of more advanced computational models and algorithms is necessary to effectively handle large-scale multi-omics data. Technologies like machine learning and artificial intelligence can be widely applied to analyze and interpret multi-omics data. This aids in discovering potential biomarkers, predicting disease risks, and providing guidance for developing personalized medicine approaches for MI. Finally, medical advancement is progressing towards intelligence, especially in the realm of precision medicine. Given the intricate nature of genes and molecules, conventional manual analysis techniques fall short of meeting requirements. The forthcoming medical paradigm will lean on intelligent models fueled by data and guided by knowledge, facilitating swifter and more precise analysis of intricate multi-omics data. These models will support physicians in rendering accurate diagnoses and treatment determinations. Particularly in the case of complex ailments like MI, the implementation of intelligent models can assist medical professionals in enhancing their comprehension of disease mechanisms and devising tailored treatment approaches.
